# Prospective associations of diabetes with 15 cancers in 2.2 million UK and Chinese adults

**DOI:** 10.1093/jnci/djaf154

**Published:** 2025-07-17

**Authors:** Bowen Liu, Sarah Floud, Ling Yang, TienYu Owen Yang, Huaidong Du, Pang Yao, Kezia Gaitskell, Yiping Chen, Jun Lv, Canqing Yu, DianJianYi Sun, Junshi Chen, Pei Pei, Liming Li, Derrick A Bennett, Zhengming Chen, Gillian K Reeves

**Affiliations:** Clinical Trial Service Unit and Epidemiological Studies Unit, Nuffield Department of Population Health, University of Oxford, Oxford, United Kingdom; Cancer Epidemiology Unit, Nuffield Department of Population Health, University of Oxford, Oxford, United Kingdom; Cancer Epidemiology Unit, Nuffield Department of Population Health, University of Oxford, Oxford, United Kingdom; Clinical Trial Service Unit and Epidemiological Studies Unit, Nuffield Department of Population Health, University of Oxford, Oxford, United Kingdom; Cancer Epidemiology Unit, Nuffield Department of Population Health, University of Oxford, Oxford, United Kingdom; Clinical Trial Service Unit and Epidemiological Studies Unit, Nuffield Department of Population Health, University of Oxford, Oxford, United Kingdom; Clinical Trial Service Unit and Epidemiological Studies Unit, Nuffield Department of Population Health, University of Oxford, Oxford, United Kingdom; Cancer Epidemiology Unit, Nuffield Department of Population Health, University of Oxford, Oxford, United Kingdom; Clinical Trial Service Unit and Epidemiological Studies Unit, Nuffield Department of Population Health, University of Oxford, Oxford, United Kingdom; Department of Epidemiology and Biostatistics, School of Public Health, Peking University Health Science Center, Beijing, China; Peking University Center for Public Health and Epidemic Preparedness & Response, Beijing, China; Ministry of Education, Key Laboratory of Epidemiology of Major Diseases (Peking University), Beijing, China; Department of Epidemiology and Biostatistics, School of Public Health, Peking University Health Science Center, Beijing, China; Peking University Center for Public Health and Epidemic Preparedness & Response, Beijing, China; Ministry of Education, Key Laboratory of Epidemiology of Major Diseases (Peking University), Beijing, China; Department of Epidemiology and Biostatistics, School of Public Health, Peking University Health Science Center, Beijing, China; Peking University Center for Public Health and Epidemic Preparedness & Response, Beijing, China; Ministry of Education, Key Laboratory of Epidemiology of Major Diseases (Peking University), Beijing, China; China National Center for Food Safety Risk Assessment, Beijing, China; Peking University Center for Public Health and Epidemic Preparedness & Response, Beijing, China; Department of Epidemiology and Biostatistics, School of Public Health, Peking University Health Science Center, Beijing, China; Peking University Center for Public Health and Epidemic Preparedness & Response, Beijing, China; Ministry of Education, Key Laboratory of Epidemiology of Major Diseases (Peking University), Beijing, China; Clinical Trial Service Unit and Epidemiological Studies Unit, Nuffield Department of Population Health, University of Oxford, Oxford, United Kingdom; Clinical Trial Service Unit and Epidemiological Studies Unit, Nuffield Department of Population Health, University of Oxford, Oxford, United Kingdom; Cancer Epidemiology Unit, Nuffield Department of Population Health, University of Oxford, Oxford, United Kingdom

## Abstract

**Background:**

Diabetes has been associated with the risk of numerous cancers, but the causal relevance of many of these associations remains unclear.

**Methods:**

We investigated associations between diabetes and risks of 15 cancers using Cox-regression and individual-level data from 2.2 million adults (334 978 incident cancer cases) in 3 prospective cohorts, UK Biobank, Million Women Study, and China Kadoorie Biobank. The potential impact of residual confounding was assessed by examining changes in diabetes-associated log hazard ratios (HRs) after adjustment for key confounders.

**Results:**

In combined analyses of individual participant data from 3 studies, diabetes was positively associated with the risk of 11 cancers, most notably liver (HR = 2.04, 95% CI = 1.87 to 2.23), pancreas (HR = 1.62, 95% CI = 1.48 to 1.77), and bladder (HR = 1.44, 95% CI = 1.29 to 1.62) cancer. The positive associations of diabetes with cancers of the breast, endometrium, kidney, and esophageal adenocarcinoma were substantially attenuated (>50%) after adjustment for confounders. The risks were similar in UK and Chinese populations except for liver cancer for which the adjusted hazard ratio was greater in UK than Chinese adults (HR = 2.58, 95% CI = 2.28 to 2.92, vs HR = 1.61, 95% CI = 1.43 to 1.83; P_het_ = 2.5 x 10^−6^). For liver cancer, the excess risk associated with diabetes increased with increasing body mass index (*P*_trend_ = 2.7 x 10^−4^) and alcohol intake (*P*_trend_ = .02). Diabetes was inversely associated with incidence of prostate cancer (HR = 0.78, 95% CI = 0.73 to 0.85) but positively associated with mortality (HR = 1.25, 95% CI = 1.00 to 1.55).

**Conclusions:**

Diabetes increases the risk of liver, pancreatic, and bladder cancer in UK and Chinese populations. It may also have a lesser effect on stomach, colorectal cancer, and leukemia, but its associations with other cancers could well be explained by confounding and/or other biases.

## Introduction

Type 2 diabetes affects more than 480 million adults worldwide, and its prevalence is increasing in most countries.^1^ Individuals with diabetes are at increased risk of cardiovascular, renal, neurological, and infectious diseases,^2^ and of more than 10 different types of cancer.^3^ For cancer, however, uncertainty remains about the extent to which diabetes is an independent risk factor, given that diabetes shares several risk factors with many cancers,^4^ including adiposity, alcohol intake, and smoking. Few studies have properly evaluated the robustness of these associations to residual confounding using individual-level data, or the impact of reverse causation bias, which could arise if preclinical symptoms of cancer were to affect the development or ascertainment of diabetes.

Most evidence on diabetes and cancer risk comes from Western populations, with limited information from regions like China, where the prevalence, detection, and treatment rates of diabetes, cancer incidence rates, and patterns of lifestyle and other factors (eg, adiposity) differ substantially. Because the confounding structure for the association of diabetes with cancer may vary importantly by region, direct comparison of associations between diabetes and cancer in the UK and China may help clarify which associations are most likely to be causal. Moreover, because diabetes is more commonly undiagnosed in China,^5^ prospective studies in China which screen for diabetes at recruitment allow for assessment of the impact on diabetes-cancer associations of differential case ascertainment and of antidiabetic medications and management among diagnosed patients.

We present detailed analyses of the association of diabetes with the risk of 15 cancers among 2.2 million adults from 3 large prospective studies in the UK and China. The main aims of the study were to (1) examine the associations of diabetes with incidence of, and mortality from, 15 cancers in UK and Chinese populations; (2) investigate whether the associations vary by method of diabetes ascertainment, geographical region, and key participant characteristics; and (3) assess the robustness of such associations to potential biases including residual confounding and reverse causality.

## Methods

### Study population and baseline data collection

This study includes data from participants of 2 large population-based prospective studies in the UK and 1 in China, namely the UK Biobank (UKB; *n* = 502 536), the Million Women Study (MWS; *n* = 1 321 076), and the China Kadoorie Biobank (CKB; *n* = 512 724). Detailed information about study design, study population, and data collection for these studies has been reported previously^6-8^ (Supplementary methods). At the baseline assessment, participants in all 3 studies completed questionnaires covering information on sociodemographic, anthropometric, lifestyle factors, and family and personal medical history, including prior clinical diagnosis of diabetes. In UKB and CKB, blood glucose levels were also measured, along with HbA1c in UKB. Participants in UKB and CKB were defined as having screen-detected diabetes if they had a random blood glucose level of at least 7.0 mmol/L at 8 or more hours fasting or at least 11.1 mmol/L at less than 8 hours fasting; a fasting glucose level of at least 7.0 mmol/L; or HbA1c level of at least 48 mmol/mol. Any diabetes (ie, self-reported or screen-detected diabetes) was used as the primary definition of diabetes in these analyses.

All participants of the 3 cohorts provided written informed consent. UKB and MWS were approved by the North West Multi-Centre Research Ethics Committee and the East of England—Cambridge South Research Ethics Committee, respectively. CKB received ethical approvals from the Oxford University Tropical Research ethics committee and the Chinese Centre for Disease Control and Prevention ethical review committee.

### Outcome measures

All 3 cohorts were followed up for incident cancer through electronic linkage to national health records (Supplementary methods). We restricted the analyses to 14 anatomic cancer sites, each with at least 300 cases among participants with prevalent diabetes across the 3 cohorts, to ensure sufficient statistical power for reliable assessment of associations. Given the known differences in etiology of esophageal cancer subtypes, esophageal cancers were subclassified by histological subtype. In UK studies, this classification was based on the *International Classification of Diseases for Oncology* (*ICD-O*) morphology coding (adenocarcinoma: *ICD-O*: 8140, 8144, 8145, 8480, 8481, 8490; squamous cell carcinoma: *ICD-O*: 8070-8073).^9^ In CKB, only approximately 5% of a subset of histologically confirmed esophageal cancers were adenocarcinomas, so all esophageal cancers in CKB were treated as squamous cell carcinomas. The main outcome of interest was first occurrence of each of 15 cancers (including 2 forms of esophageal cancer; Table S1). In addition to incidence, mortality from each cancer was analyzed separately.

### Statistical analyses

Participants were excluded from all analyses if they had a prior cancer diagnosis (other than nonmelanoma skin cancer [*ICD-10* C44] in UKB and MWS) or missing information on diabetes or body mass index (BMI) at recruitment, and were excluded from MWS analyses if they had also participated in UKB. In analyses of endometrial and ovarian cancer, women with a history of hysterectomy or bilateral oophorectomy (any oophorectomy in CKB), respectively, were also excluded.

Person-years were calculated from baseline to the first of any malignant cancer (*ICD-10* C00-C97, other than nonmelanoma skin cancer in UKB and MWS); death from any cause; emigration or other loss to follow-up; or end of follow-up for cancer (or for death in mortality analyses).

Cox regression models were used to estimate hazard ratios (HRs) for cancers associated with any diabetes. The main analyses were stratified by birth cohort (5-year categories of year of birth), sex, and geographical region and adjusted simultaneously for the following: model 1—socioeconomic factors (highest educational qualification in all 3 cohorts, and ethnicity and quintiles of Townsend deprivation index in UKB and MWS); model 2—all factors included in model 1 plus smoking status, alcohol consumption level, physical activity, adiposity and early life adiposity traits, history of hypertension and blood pressure (systolic and diastolic), and reproductive factors (in women only). All factors in model 2 were selected based on previous prospective evidence of their associations with risks of cancers and diabetes.^10-24^ Adiposity traits in model 2 included continuous BMI in all cohorts and continuous waist circumference and categorical early life adiposity traits in UKB and CKB. Early life adiposity traits in UKB and CKB included self-reported comparative body size at age 10 years in UKB (thinner, plumper than average, or about average) and quintiles of self-reported weight at age 25 years in CKB. Smoking status was classified as current smoker of at least 15 cigarettes per day, current smoker of less than 15 cigarettes per day, former smoker, occasional smoker (UKB and CKB only), and never-smoker. Alcohol consumption was classified in UKB and CKB, according to average consumption, as at least 2 units per day, 1-2 units per day, weekly with less than 1 unit per day, monthly, occasional, previous regular, and never regular. In MWS, alcohol consumption was classified as at least 2 units per day, 1-2 units per day, weekly with less than 1 unit per day, monthly (<1 drink/week), and nondrinker. Details of adjustments for other factors are summarized in the Supplementary Methods. An inverse variance-weighted meta-analysis was conducted to combine results from UKB and MWS and from all 3 cohorts.

We assessed associations according to certain characteristics of the population, including sex, age at risk (younger than 65, 65-74, 75 years and older), BMI (<25, 25-30, >30 kg/m^2^); smoking status (current, past, or never regular [including individuals who smoked only occasionally but never regularly]); regular alcohol consumption (yes or no); and duration of prevalent diabetes (<3, 3-6, ≥6 years) in CKB and UKB. We further assessed associations by study locality (urban or rural) in CKB only, given the large urban-rural disparities in socioeconomic factors, lifestyle, and health-care resources across China.^6^

To help assess the risk of bias from residual confounding, we undertook individual adjustment for each covariate, with the impact of adjustment for specific covariates on the associations quantified as the corresponding percentage change in the log hazard ratio associated with diabetes.^25^^,^^26^ We also calculated *E-*values for the fully adjusted hazard ratios and for the limits of the confidence intervals (CI) closer to the null, as measures of their robustness to the effects of uncontrolled confounding.^27^^,^^28^

We examined whether there was evidence of reverse causation by repeating the main analyses by follow-up period (<5, ≥5 years). Where possible, we also compared findings using differing definitions of diabetes (self-reported, screen detected), with and without exclusion of death certificate–only cancer records for cancer incidence in CKB, and with and without exclusion of participants in CKB and UKB who reported being diagnosed with diabetes younger than age 30 years, as these were more likely to have type 1 diabetes rather than type 2 diabetes.

We used Cochrane *Q* tests or trend tests, as appropriate, to assess heterogeneity in estimated associations by country, study within country (UKB vs MWS), sex, and other characteristics. All *P* values were corrected for multiple testing using the Benjamini–Hochberg method (5% false-discovery rate) applied separately to UK cohorts, CKB, and all cohorts combined.

## Results

**Figure 1.**
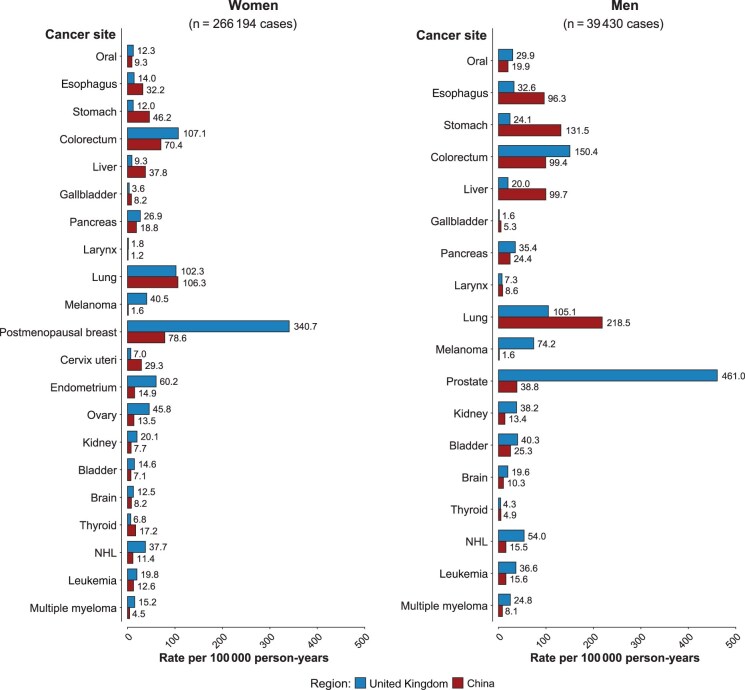
Age-standardized incidence rates of cancers after age 50 years by sex in UK and Chinese studies. Standardized by the 5-year age groups of 2021 world population structure. Abbreviation: NHL = non-Hodgkin lymphoma.

After exclusions, 2 184 391 (91.8%) participants were included in the main analyses, including 471 444 (93.8%) in UKB, 1 202 850 (88.2%) in MWS, and 510 097 (99.5%) in CKB, and the prevalence of diabetes at baseline was 5.9%, 2.7% (self-reported only), and 5.9%, respectively (Table 1). In UKB and CKB, 12.1% and 46.7% of the participants with diabetes were screen detected, respectively. During a median follow-up period of 10.8, 21.4, and 12.0 years in UKB, MWS, and CKB, respectively, there was a total of 334 978 incident cancers (UKB: 48 649; MWS: 253 612; CKB: 32 717). For all cancers, except esophageal cancer subtypes, there were at least 300 participants in those with any diabetes across all 3 cohorts, yielding adequate (≥80%) statistical power to detect even modest diabetes-associated hazard ratios (approximately 1.2) (Table S1). The incidence of different cancers varied between the UK and China; for example, incidence of breast and prostate cancer was generally higher in the UK studies, while the incidence of stomach and liver cancer was greater in CKB (Figure 1).

**Table 1.** Baseline characteristics of participants in UKB, MWS, and CKB, by diabetic status at baseline

Characteristics^a^	UKB		MWS		CKB	
	Diabetes (*n* = 27 761)	No diabetes (*n* = 443 683)	Diabetes (*n* = 32 596)	No diabetes (*n* = 1 170 254)	Diabetes (*n* = 30 004)	No diabetes (*n* = 480 093)
Sociodemographic and lifestyle factors						
Age, mean (SD), y	59.6 (7.6)	56.7 (8.2)	58.2 (4.9)	56.7 (4.9)	58.1 (10.0)	51.7 (10.8)
Female, %	40.5	54.7	100	100	58.6	58.7
College or university degree, %	26.0	34.1	8.4	12.2	7.8	6.1
Current regular smoker, %						
Women	10.2	10.5	13.9	14.5	2.5	2.5
Men	10.4	10.6	—	—	56.6	62.3
Past smoker, %						
Women	33.3	34.3	32.7	29.0	1.1	1.0
Men	34.0	34.1	—	—	16.5	12.1
Regular alcohol consumption, %						
Women	55.3	77.4	40.2	61.7	1.3	2.2
Men	72.9	87.8	—	—	34.8	33.9
Physical activity						
Total, mean (SD),^b^ MET-h/day	5.3 (12.5)	6.4 (10.9)	—	—	18.5 (18.1)	21.6 (13.6)
Weekly strenuous exercise, %	—	—	23.4	37.7	—	—
Anthropometry and physical measurements, mean (SD)						
Standing height, cm	167.9 (11.0)	169.3 (9.9)	161.2 (15.8)	161.8 (15.6)	160.5 (8.4)	159.6 (6.2)
Body weight, kg	89.1 (30.3)	78.0 (20.1)	76.2 (31.1)	67.9 (29.7)	59.1 (14.2)	56.0 (7.9)
Body mass index, kg/m^2^	31.6 (10.4)	27.2 (6.7)	29.3 (11.6)	25.9 (10.9)	25.2 (5.1)	23.5 (3.4)
Waist circumference, cm	101.2 (24.3)	89.7 (17.2)	—	—	85.9 (13.7)	80.3 (9.8)
Waist-hip ratio	0.93 (0.12)	0.87 (0.10)	—	—	0.92 (0.09)	0.88 (0.07)
Systolic blood pressure, mmHg	138.5 (26.5)	136.3 (28.1)	—	—	138.1 (26.7)	130.8 (19.9)
Diastolic blood pressure, mmHg	82.6 (16.9)	82.2 (15.5)	—	—	80.8 (15.7)	77.8 (11.3)
Random blood glucose, mmol/L	7.8 (6.6)	4.9 (1.1)	—	—	12.4 (7.8)	5.7 (1.2)
Medical history, %						
Heart disease, %	5.6	1.7	19.0	7.2	5.8	2.7
Stroke,^c^ %	2.8	1.1	5.3	1.6	3.5	1.6
Hypertension, %	53.9	23.0	64.3	40.2	22.1	10.6
Family history of diabetes, %	47.0	20.7	—	—	21.0	6.2

a All baseline characteristics in 3 studies were adjusted for the 2021 world population structure (United Nations) by 5-year age groups and sex.

b In CKB, this included work-related and leisure time physical activity, and in 2 UK studies, it included only leisure time physical activity.

c In CKB, this referred to self-reported physician-diagnosed history of stroke or transient ischemic attack.

Em dashes indicate unavailable information in the specific study. Abbreviations: CKB = China Kadoorie Biobank; MWS = Million Women Study; UKB = UK Biobank; WC = waist circumference.

**Figure 2.**
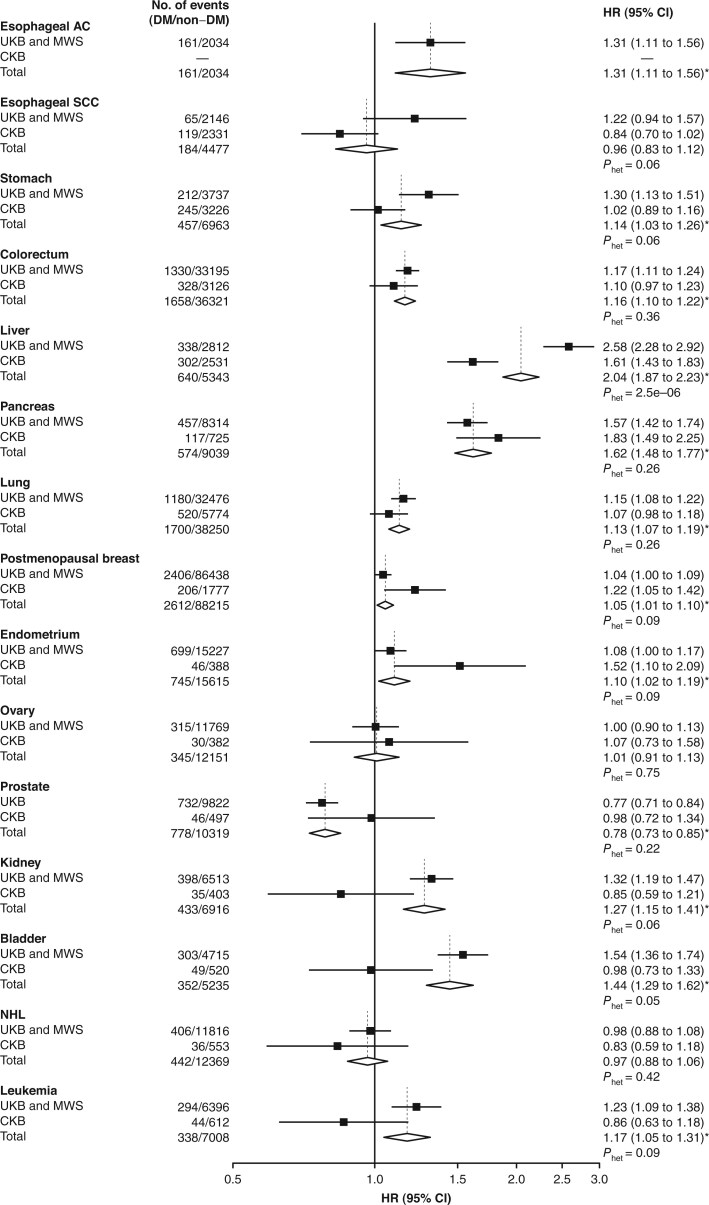
Associations of diabetes with risks of 15 cancers in UK and Chinese studies. Cox regression models comparing participants with vs without diabetes with attained age as the underlying time variable, stratified by year of birth, region and sex, and adjusted for socioeconomic factors, smoking, alcohol, physical activity, adiposity, hypertension, blood pressure, and female reproductive factors. Each **solid square** represents the estimated hazard ratio. The **horizontal lines** indicate 95% confidence intervals. Hazard ratios for UKB and MWS are the combined results of the cohort-specific hazard ratios from UKB and MWS in fixed-effect meta-analysis, and the overall hazard ratios are the combined results of all 3 cohorts, which are represented by the **diamonds**. Esophageal AC and esophageal SCC indicate adenocarcinoma and squamous cell carcinoma of esophagus, respectively. Number of events are the number of participants with cancer in those with or without prevalent diabetes at baseline. **Em dashes** for esophageal AC in CKB indicate that this analysis was not conducted in CKB. *P*_het_ indicates *P* values for heterogeneity test after adjustment for FDR at 0.05. Cancers with FDR-adjusted statistically significant *P* value (<0.05) in the meta-analysis of all 3 studies are marked with an asterisk on the hazard ratio (95% CI). Abbreviations: CI = confidence interval; CKB = China Kadoorie Biobank; DM = diabetes mellitus; FDR = false discovery rate; HR = hazard ratio; MWS = Million Women Study; NHL = non-Hodgkin lymphoma; UKB = UK Biobank.

In combined analyses of the UK studies, diabetes was associated with statistically significant increases in the risks of 9 cancers, with adjusted hazard ratios ranging from 1.15 (95% CI = 1.08 to 1.22) for lung cancer to 2.58 (95% CI = 2.28 to 2.92) for liver cancer (Figure S1). In CKB, the associations of diabetes with most cancers were broadly similar to those in UK studies, at least directionally (Figure 2). In combined analyses of all 3 studies, diabetes was associated with increased risks of 11 cancers, with the highest adjusted hazards ratios for liver (HR = 2.04, 95% CI = 1.87 to 2.23), pancreatic (HR = 1.62, 95% CI = 1.48 to 1.77), and bladder (HR = 1.44, 95% CI = 1.29 to 1.62) cancer, and more modest positive associations (HRs ranging from 1.05 to 1.31) for esophageal adenocarcinoma, stomach, colorectal, lung, postmenopausal breast, endometrial, and kidney cancer, and leukemia (Figure 2). There was a statistically significant inverse association of diabetes with prostate cancer risk (HR = 0.78, 95% CI = 0.73 to 0.85), driven mainly by UKB data.

There was no statistically significant heterogeneity in the adjusted hazard ratios for cancers by method of diabetes ascertainment (self-report vs screen detected) (Figure S2), by sex (Figure S3), or between UK and Chinese populations (Figure 2), except for liver cancer for which the adjusted hazard ratio was significantly greater in UK than in Chinese populations (HR = 2.58, 95% CI = 2.28 to 2.92, vs HR = 1.61, 95% CI = 1.43 to 1.83; *P*_het_ = 2.5 x 10^−6^), and this difference was evident in both men (HR = 3.24, 95% CI = 2.53 to 4.13, vs HR = 1.62, 95% CI = 1.39 to 1.90; *P*_het_ = 3.9 x 10^−5^) and women (HR = 2.37, 95% CI = 2.05 to 2.75, vs HR = 1.60, 95% CI = 1.31 to 1.96; *P*_het_ = 2.1 x 10^−3^). By contrast, the estimated absolute difference in the risk of liver cancer associated with diabetes was much greater in the Chinese than in UK populations because of higher underlying rates of liver cancer in Chinese populations, with an estimated 33.6 (95% CI = 29.1 to 38.0) extra liver cancer cases per 100 000 person-years in Chinese populations (HR = 88.6, 95% CI = 84.8 to 92.3, in participants with diabetes, vs HR = 55.0, 95% CI = 52.7 to 57.3, without diabetes) compared with 14.8 (95% CI = 11.0 to 18.6) in UK populations (HR = 24.1, 95% CI = 20.6 to 27.7, vs HR = 9.4, 95% CI = 8.0 to 10.7).

Associations of diabetes with mortality from specific cancers were broadly similar to corresponding associations with incidence except for prostate cancer. In contrast to the approximately 20% lower risk of prostate cancer observed in men with diabetes, there was weak evidence of a modest increase in mortality from prostate cancer in men with diabetes (HR = 1.25, 95% CI = 1.00 to 1.55) (Figure S4).

**Figure 3.**
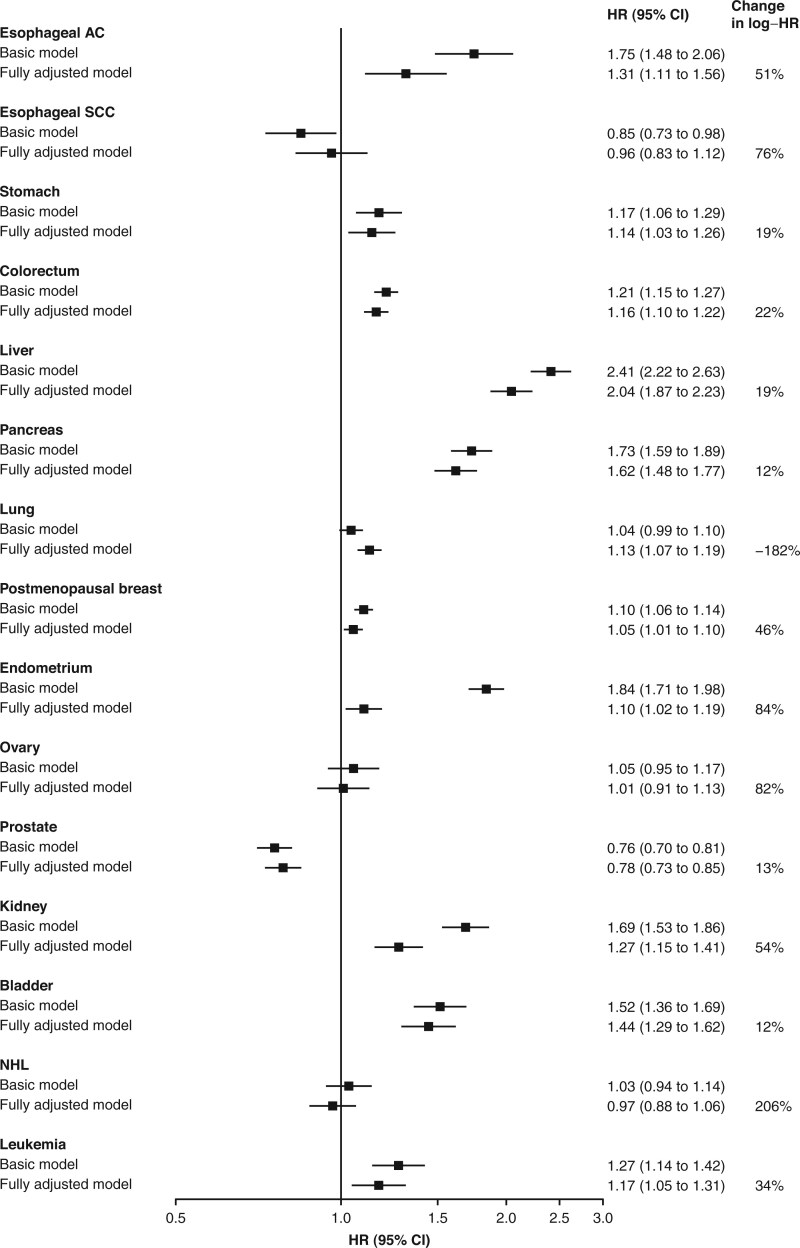
Associations of diabetes with risks of 15 cancers in UK and Chinese studies by level of adjustment. Cox regression comparing associations between diabetes and risk of cancers in the basic model with adjustment for socioeconomic factors only (highest education in all 3 cohorts and Townsend Deprivation index and ethnicity in UKB and MWS) with those in the fully adjusted model. Changes in the estimated associations are calculated by taking the proportional difference between the fully adjusted log hazard ratio and the log hazard ratio from the basic model. Abbreviations: AC = adenocarcinoma; CI = confidence interval; HR = hazard ratio; MWS = Million Women Study; NHL = non-Hodgkin lymphoma; SCC = squamous cell carcinoma; UKB = UK Biobank.

Figure 3 shows the proportional changes in the log hazard ratios between basic and fully adjusted models. With the exception of lung cancer, the hazard ratios for all cancers were attenuated to varying degrees after full adjustment, with the most marked attenuations observed for endometrial cancer (by 84%), kidney cancer (54%), esophageal adenocarcinoma (51%), and postmenopausal breast cancer (46%). In most cases, this attenuation was driven largely by adjustment for adiposity-related metrics, but for kidney cancer, adjustment for hypertension also had a notable impact (Figure S5). In contrast, the hazard ratio for lung cancer increased from 1.04 to 1.13 after full adjustment, which was driven mainly by adjustment for smoking and adiposity-related metrics (Figure 3;  Figure S5). The *E-*values for the hazard ratios of liver, pancreatic, and bladder cancers were more than 2, while for stomach, colorectal cancer, and leukemia, they were all more than 1.5 (Table S2).

**Figure 4.**
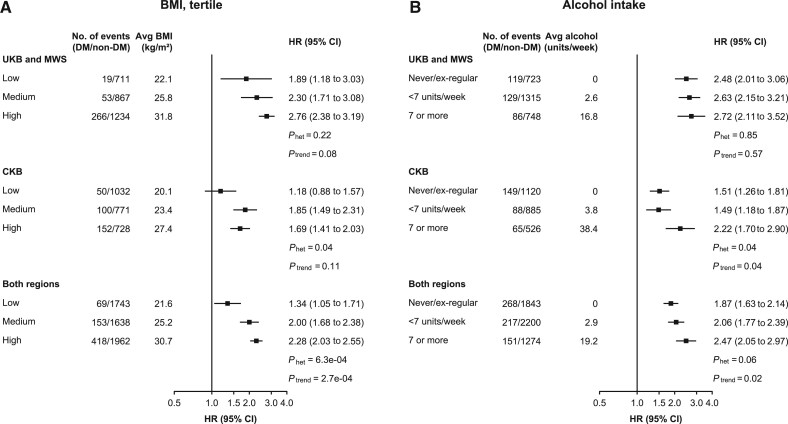
Associations of diabetes with risk of liver cancer in UK and Chinese studies by levels of (**A**) BMI and (**B**) alcohol intake. *The average alcohol intake level in the subgroup of CKB with less than 7 units per week was calculated within those who drank alcohol every week. P_het_ and P_trend_ indicate P values for heterogeneity test and trend test. Abbreviations: avg = average; BMI = body mass index; CI = confidence interval; CKB = China Kadoorie Biobank; DM = diabetes mellitus; HR = hazard ratio; MWS = Million Women Study; UKB = UK Biobank.

The main findings for all 3 studies combined were broadly similar by age at risk (Figure S6) and by region of residence in China (urban vs rural) (Figure S7), although there was weak evidence of a smaller association with prostate cancer and a greater association with bladder cancer, with increasing age (*P*_trend_ = .03 for both cancers). The association of diabetes with individual cancers was also similar in subgroups of participants defined by smoking status, BMI, and alcohol intake (Figures S8-S10). The only exceptions to this were for liver cancer, for which associations with diabetes were greater with higher BMI (*P*_trend_ = 2.7 x 10^−4^) (Figure 4, A) and with higher alcohol intake (*P*_trend_ = .02) (Figure 4, B). While the diabetes-associated lung cancer risk did not vary materially by smoking status, there was no clear evidence of an association in never smokers (HR = 1.11, 95% CI = 0.99 to 1.24) (Figure S8).

There was no evidence of variation in adjusted hazard ratios for cancers by follow-up period, except for liver cancer (Figure S11) for which the hazard ratio was greater in the period 5 or more years than in the first 5 years after baseline (HR = 2.31, 95% CI = 2.09 to 2.56, vs HR = 1.46, 95% CI = 1.22 to 1.74; *P*_het_ = 1.5 x 10^−4^). Further sensitivity analyses showed no statistically significant differences in hazard ratios by self-reported duration of diabetes at baseline (Figure S12), after exclusion of participants with incident cancer recorded from death certificate only in CKB (Figure S13), or after excluding diabetes diagnosed before the age of 30 years in UKB and CKB (Figure S14).

## Discussion

Our analysis of 2.2 million participants from the UK and China provides new evidence of robust associations of diabetes with increased risks of liver, pancreas, and bladder cancer and possible, albeit modest, excess risks of stomach, and colorectal cancer, and leukemia, after taking careful account of the likely impacts of residual confounding and other biases. Our findings also provide evidence that previously reported associations of diabetes with several other cancers are likely to have been materially influenced by residual confounding or ascertainment bias. With the exception of liver cancer, the observed associations were similar regardless of study population (the UK vs China), sex, method of diabetes ascertainment, and follow-up period.

Our findings align with previous prospective studies and meta-analyses reporting positive associations of diabetes with risks of more than 10 site-specific cancers, although few such studies were able to adequately adjust for all relevant confounders or assess the impact of potential biases on their results.^4^ By assessing the impacts of adjustment for confounders using individual-level data with extensive follow-up and detailed health-related information at recruitment, we demonstrated that the positive associations of certain cancers (liver, pancreas, bladder, colorectal, stomach, leukemia) with diabetes are reasonably robust to adjustment for confounding. However, the associations of diabetes with several other cancers, particularly endometrial cancer, could be due toinadequate adjustment for important confounders such as BMI and/or smoking.

The strongest and most robust association observed here was between diabetes and liver cancer, which was consistent with previous prospective studies, meta-analyses, and Mendelian randomization evidence in populations with European ancestry.^29-31^ We also found a highly statistically significant disparity in the association of diabetes with liver cancer between UK and Chinese populations. The smaller diabetes-associated HR for liver cancer observed in Chinese populations may be explained by differences in underlying causes and rates of liver cancer, such as the high proportion of adults infected by hepatitis B and C virus in China,^32^ leading to a fourfold greater incidence of liver cancer compared with the UK (Figure 1). Nevertheless, the absolute excess risk associated with diabetes was twice as high in Chinese (33.6 per 100 000) as in UK (14.8 per 100 000) populations. We also found a greater association of diabetes with liver cancer among individuals with higher BMI, and those with higher alcohol intake, which has not been previously reported. This is biologically plausible given that nonalcoholic fatty liver disease is strongly related to adiposity and diabetes and increases the risk of liver cancer.^33^ However, further replication in other large prospective or genetic studies is needed to confirm this apparent effect modification.

Our finding of a positive association of diabetes with pancreatic cancer is consistent with those of previous prospective studies, meta-analyses, and Mendelian randomization studies in populations of European ancestry,^34-37^ although our hazard ratio of 1.62 was slightly lower than the pooled estimate of 1.88 from a meta-analysis of 50 prospective studies.^35^ It has been suggested that new onset diabetes may be a manifestation of undiagnosed or early stage pancreatic cancer,^38^ but the fact that diabetes-associated risks for pancreatic cancer in our data did not vary materially by follow-up period does not support this.

For stomach, colorectal, and bladder cancer, and leukemia, our results also align with previous meta-analyses, although for bladder cancer the effect size from the latest meta-analysis of 30 prospective studies was somewhat smaller than the present study (RR = 1.23 vs HR = 1.44).^3^^,^^39^ It is conceivable that associations of diabetes with leukemia risk might be affected by ascertainment bias, because of, for example, increased blood testing among diabetes patients.^40^ However, we found no evidence of heterogeneity in the association with leukemia risk by time since diabetes diagnosis, and we observed similar associations of diabetes with leukemia incidence and mortality, suggesting that ascertainment bias is unlikely to have materially affected these findings.

In the present study, the diabetes-associated log hazard ratios for esophageal adenocarcinoma, kidney, and postmenopausal breast cancer were considerably (∼50%), and for endometrial cancer, almost entirely (∼85%), attenuated after adjustment for confounders, particularly BMI and/or hypertension, suggesting that these findings may be subject to residual confounding.^25^^,^^26^ Notably, our combined analyses observed only a 9% excess risk of endometrial cancer after accounting for key confounders, which was much smaller than the approximate 60% excess risk reported by 2 independent meta-analyses of prospective studies,^3^^,^^41^ highlighting the importance of thorough adjustments for confounders. Although the association of diabetes with lung cancer was not attenuated after adjustment for smoking and other potential confounders, information on smoking intensity in past smokers was limited, and smoking habits are likely to be measured with some degree of error. The absence of an association in never regular smokers (Figure S8), consistent with previous prospective findings,^42^ suggests that the small excess risk of lung cancer is unlikely to be causal.

For prostate cancer, we found an inverse association of diabetes with the risk of incidence, consistent with previous findings,^43^ but a weak positive association with the risk of mortality. The lower levels of prostate-specific antigen in men with diabetes may reduce the likelihood of referral for further investigation, leading to lower detection rates of less aggressive prostate cancer^44-46^ but not necessarily lower mortality. The inverse association with diabetes observed here is, therefore, more likely to reflect differential screening sensitivity in diabetes patients rather than a causal relationship.

The present study has several strengths including considerable statistical power for most cancers, prolonged follow-up within each cohort, and comprehensive information on related risk factors, enabling a detailed assessment of the impacts of residual confounding and reverse causation on the findings. However, the study also has some limitations. Firstly, there were insufficient cases to study rarer cancers such as gallbladder cancer, which has previously been associated with diabetes, largely on the basis of retrospective or routine record linkage studies. Secondly, we cannot exclude the possibility that those with diabetes may be more likely be diagnosed earlier with cancer because of increased medical surveillance following a diabetes diagnosis, although this is unlikely to represent a major bias. Thirdly, because we did not have specific information on type of diabetes, we were unable to directly assess the specific effects of type 1 diabetes and type 2 diabetes on cancer risk. However, given that type 2 diabetes generally makes up the vast majority (>95%^1^) of diabetes cases and that exclusion of individuals diagnosed with diabetes at a relatively young age in UKB and CKB yielded similar results, the observed findings can be assumed to largely reflect the effects of type 2 diabetes on cancer risk.

In summary, our findings highlight that diabetes is strongly associated with liver, pancreas, and bladder cancer and may also be related to stomach cancer, colorectal cancer, and leukemia, however, previously reported associations for several other cancers may not be directly attributable to diabetes itself. Preventing diabetes, through adiposity control or other strategies, could substantially reduce the risks of certain cancers. Further investigations should aim to elucidate the mechanisms by which diabetes affects these cancers, to guide prevention and screening strategies and inform targeted interventions.

## Supplementary Material

djaf154_Supplementary_Data

## Data Availability

Data from CKB and the MWS are available to bona fide researchers in accordance with the CKB Data Access Policy (https://www.ckbiobank.org/data-access/data-2) and the MWS Data Access Policy (https://www.ceu.ox.ac.uk/research/the-million-women-study/data-access-and-sharing/data-access-policy), respectively. This research has been conducted using the UKB Resource under application number 67506. The data underlying this article will be shared on reasonable request to the corresponding author.

## Members of the CKB Collaborative Group

International Steering Committee: Junshi Chen, Zhengming Chen (PI), Robert Clarke, Rory Collins, Liming Li (PI), Jun Lv, Richard Peto, Robin Walters. International Co-ordinating Centre, Oxford: Daniel Avery, Maxim Barnard, Derrick Bennett, Ruth Boxall, Ka Hung Chan, Yiping Chen, Zhengming Chen, Jonathan Clarke; Robert Clarke, Huaidong Du, Ahmed Edris Mohamed, Hannah Fry, Simon Gilbert, Prapthi Harish, Pek Kei Im, Andri Iona, Maria Kakkoura, Christiana Kartsonaki, Kshitij Kolhe, Hubert Lam, Kuang Lin, James Liu, Iona Millwood, Sam Morris, Qunhua Nie, Alfred Pozarickij, Maryam Rahmati, Paul Ryder, Dan Schmidt, Becky Stevens, Iain Turnbull, Robin Walters, Baihan Wang, Lin Wang, Neil Wright, Ling Yang, Xiaoming Yang, Pang Yao.

National Co-ordinating Center, Beijing: Jun Lv, Canqing Yu, Dianjianyi Sun, Yuanjie Pang, Can Hou, Qingmei Xia, Chao Liu, Pei Pei, Lang Pan, Xiao Han, Honglu Bian, Xinxin Chen.

Ten Regional Co-ordinating Centers: Qingdao CDC: Zengchang Pang, Ruqin Gao, Shanpeng Li, Haiping Duan, Shaojie Wang, Yongmei Liu, Ranran Du, Liang Cheng, Xiaocao Tian, Hua Zhang. Licang CDC: Dan Hu, Xiaoyan Zheng, Yujie Wang. Heilongjiang Provincial CDC: Wei Sun, Shichun Yan, Xiaoming Cui. Nangang CDC: Chi Wang, Zhenyuan Wu, Lishun Zhai, Zhaoxi Pang, Shiwen Dong. Hainan Provincial CDC: Huiming Luo, Jinyan Chen, Bin He, Dingwei Sun, Xingren Wang, Tingting Ou. Meilan CDC: Xiangyang Zheng, Dewei Zheng, Shuai Yang, Yilei Li, Lihui Li, Xingjiao Chen. Jiangsu Provincial CDC: Jinyi Zhou, Ran Tao, Jian Su, Xikang Fan, Zongming Cheng, Yuxiao Huang. Suzhou CDC: Yan Lu, Yujie Hua, Li Xing, Shuxian Wang, Jianrong Jin, Juping Ma, Jinchao Liu, Kaifei Zhu, Hongfu Ren, Xingfeng Shen. Guangxi Provincial CDC: Ge Zhong, Wei Mao, Zhenzhen Lu, Ling He. Liuzhou CDC: Lifang Zhou, Changping Xie, Jian Lan, Tingping Zhu, Jinxue Tan, Liuping Wei, Liyuan Zhou, Sisi Wang. Sichuan Provincial CDC: Xianping Wu, Ningmei Zhang, Xiaofang Chen, Xiaoyu Chang, Zhuo Wang, Yujin He. Pengzhou CDC: Mingqiang Yuan, Xia Wu, Xiaofang Chen, Zhaodong Wang, Qiang Sun, Yang Lin. Gansu Provincial CDC: Faqing Chen, Xiaolan Ren, Lijun Chang, Feiming Zhong. Maiji CDC: Jianjun Feng, Weijie Hu, Xiaofang Zhang, Yalin Chen, Fei Wang, Jun Wang. Henan Provincial CDC: Linqi Diao, Wanshen Guo, Zhiwei Han, Dongyang Zhao, Dengjun Zhu, Kai Kang, Shixian Feng, Huizi Tian, Yali Yan, Bing Han, Li Gao, Shaofang Li, Huafei Feng, Wei Tang. Huixian CDC: Xiaolin Li, Huarong Sun, Xiaocong Zhao, Ying Li, Chen Hu, Pan He, Xukui Zhang, Yuanyuan Jin, Hesheng Zhang. Zhejiang Provincial CDC: Min Yu, Ruying Hu, Hao Wang, Weiwei Gong, Jieming Zhong, Meng Wang, Chunxiao Xu, Keqing Gong. Tongxiang CDC: Hao Xu, Yuan Cao, Kaixu Xie, Lingli Chen, Xiaomei Tu, Chen Chen. Hunan Provincial CDC: Xiaojun Li, Li Yin, Huilin Liu, Yuan Liu, Yi Liu, Lei Yin, Xian Xie, Jing Wang. Liuyang CDC: Bo Xiao, Pingsheng Lou, Yuan Peng, Libo Zhang, Chan Qu, Qili Jiang, Yanling Chen, Yan Zhao.

## Membership of the MWS

Advisory committee: Emily Banks (chair), Naomi Allen, Andy Boyd, Sarah Floud, Lesley Laxton, Delyth Morgan, Julietta Patnick, Richard Peto, Gillian Reeves, Cathie Sudlow and Magdalen Wind Mozley.

Coordinating center staff: Simon Abbott, Rupert Alison, Sarah Atkinson, Krys Baker, Isobel Barnes, Judith Black, Anna Brown, Sarah Floud, Kezia Gaitskell, Toral Gathani, Laura Gerrard, Adrian Goodill, Carol Hermon, Lina Jarutyte, Sau Wan Kan, Nicky Langston, Keren Papier, Kirstin Pirie, Gillian Reeves, Keith Shaw, Emma Sherman, Karl Smith-Byrne, Helena Strange, Siân Sweetland, Ruth Travis, Lyndsey Trickett, Owen Yang, and Heather Young.
