# Supplementary material for: Prospective associations of diabetes with 15 cancers in 2.2 million UK and Chinese adults
Source: J Natl Cancer Inst. 2025 Jul 17;117(12):2477–87. doi: 10.1093/jnci/djaf154 (PMC12682376; doi:10.1093/jnci/djaf154)

Supplementary material

Prospective associations of diabetes with 15 cancers in 2.2 million UK and Chinese adults

Supplementary Methods 2
Table S1. Definitions for incident cancer outcomes and statistical power of Cox regression 4
Table S2. Assessments for residual confounding of fully adjusted associations in meta-analysis of UKB, MWS and CKB 5
Figure S1. Associations of diabetes with 15 cancers in UKB and MWS 6
Figure S2. Associations of self-reported and screen-detected diabetes with 15 cancers in UKB and CKB 7
Figure S3. Associations of diabetes with 15 cancers by sex in UKB, MWS and CKB 8
Figure S4. Associations of diabetes with 15 cancer mortality in UK and Chinese studies 9
Figure S5. Impact of adjustment for potential confounders on associations of diabetes with 15 cancers in UKB, MWS and CKB 10
Figure S6. Associations of diabetes with 15 cancers in UK and Chinese populations, by age at risk... 14
Figure S7. Associations of diabetes with 15 cancers in CKB, by region 15
Figure S8. Associations of diabetes with 15 cancers in UK and Chinese populations, by smoking status 16
Figure S9. Associations of diabetes with 15 cancers in UK and Chinese populations, by BMI 17
Figure S10. Associations of diabetes with 15 cancers in UK and Chinese populations, by alcohol consumption 18
Figure S11. Associations of diabetes with 15 cancers in UK and Chinese populations, by follow-up period 19
Figure S12. Associations of diabetes with 15 cancers in UK and Chinese populations, by self-reported duration of diabetes 20
Figure S13. Associations of diabetes with 15 cancers in CKB, with and without exclusion of death certificate only cancer registrations 21
Figure S14. Associations of diabetes with 15 cancers in UKB and CKB, before and after excluding prevalent diabetes diagnosed before age 30 22

Supplementary Methods

Study populations

UKB recruited 502,536 participants aged 40-69 years from 22 assessment centres in England, Wales and Scotland during 2006-2010. MWS recruited 1.3 million women from England and Scotland between 1996 and 2001 through the UK national breast cancer screening programme which invites all women aged 50-64 for routine mammographic screening. About 0.3% of UKB participants self-identified as Chinese. In MWS, participants were not specifically asked if they identified as Chinese, but 0.4% reported an ethnicity other than White, Black or South Asian. The CKB recruited 512,724 participants aged 30-79 from 5 urban and 5 rural geographically defined localities of China during 2004-2008. Almost all CKB participants were Han Chinese, and none identified as of European ethnicity. In each study, ethics approval was obtained from relevant international, national and regional ethics committees or institutional research boards, and all participants provided written informed consent.

Among a total of 2,305,836 participants who were free from cancer at recruitment into UKB (474,661), MWS (1,321,076) and CKB (510,099), 1,719 were excluded due to missing information on diabetes status, 69,681 were excluded due to missing information on BMI, and 50,045 women were excluded from analyses in MWS due to their participation in both MWS and UKB.

Longitudinal follow-up methods

In UKB and MWS, participants were followed up for incident cancer through electronic linkage to NHS health records. In CKB, information on cancer incidence was collected through linkages via a unique 18-digit personal ID number with cancer registries, mortality registries and any episodes of hospitalisation as part of the national health insurance system. In all three studies, ICD-10 was used to code site-specific cancers. The end of follow-up for cancer was February 29th 2020, December 31st 2016, and January 31st, 2021, respectively, for UKB participants in England, Wales and Scotland; December 31st 2020 for MWS participants; and December 31st 2018 for CKB participants. The end of follow-up for death was September 30th 2021 and October 31st 2021, respectively, for UKB participants in England/Wales and Scotland; December 31st 2021 for all MWS participants; and December 31st 2018 for CKB participants.

Details of covariates

In UKB and CKB, height, weight, waist and hip circumferences, and blood pressure, were measured using standardised protocols, while in the MWS, self-reported weight and height (but not waist circumference and blood pressure) were collected and used to calculate BMI.

Physical activity was categorised according to quintiles of MET-h/day in UKB and CKB, and self-reported weekly frequencies of any exercise and strenuous exercise (daily, 4-6, 2-3, 1, <1, rare or never) in MWS. Self-reported history of hypertension was adjusted in all cohorts, and continuous systolic and diastolic blood pressures were adjusted in UKB and CKB. Female reproductive factors included use of oral contraceptives, menopausal status (yes/no), use of menopausal hormone therapy (available in UKB and MWS only), parity, and age at menarche, age at menopause and age at first live birth. Menopausal status was categorised as pre- or post-menopausal. Use of oral contraceptives and use of menopausal hormone therapy was categorised as: current, past, or never use. Parity was categorised as: 0, 1-2, 3-4 and 5+ children. Age at menarche, age at menopause and age at first live birth were categorised according to the study-specific quintiles. Participants with missing information of these covariates were assigned to the missing category in the relevant variables. The general missing information of covariates were negligible in UKB and CKB (<3% for most variables) as the interviews were done in person via touchscreen. The proportion with missing information in MWS was generally low for most included variables collected from the baseline questionnaire (<5%).

Table S1. Definitions for incident cancer outcomes and statistical power of Cox regression

Cancer site	Definitions		UKB and MWS			CKB			All studies combined		
	ICD-10	ICD-O	No. of Cases	Power (HR=1.2)	Power (HR=1.5)	No. of Cases	Power (HR=1.2)	Power (HR=1.5)	No. of Cases	Power (HR=1.2)	Power (HR=1.5)
Oesophageal adenocarcinoma	C15	8140, 8144, 8145, 8480, 8481, 8490	2195	35.2%	94.0%	-	-	-	2195	35.2%	94.0%
Oesophageal squamous cell carcinoma	C15	8070, 8071, 8072, 8073	2211	35.5%	94.2%	2450	55.3%	99.7%	4661	68.9%	100.0%
Stomach	C16		3949	56.4%	99.7%	3471	70.3%	100.0%	7420	87.2%	100.0%
Colorectum	C18-C20		34525	100.0%	100.0%	3454	70.1%	100.0%	37979	100.0%	100.0%
Liver	C22		3150	47.4%	98.8%	2833	61.5%	99.9%	5983	79.4%	100.0%
Pancreas	C25		8771	88.5%	100.0%	842	23.2%	77.9%	9613	94.1%	100.0%
Lung	C34		33656	100.0%	100.0%	6294	91.9%	100.0%	39950	100.0%	100.0%
Postmenopausal breast	C50		88844	100.0%	100.0%	1983	48.4%	99.0%	90827	100.0%	100.0%
Endometrium	C54.1		15926	97.3%	100.0%	434	14.4%	51.5%	16360	98.9%	100.0%
Ovary	C56		12084	92.2%	100.0%	412	13.9%	49.5%	12496	96.0%	100.0%
Prostate	C61		10554	99.9%	100.0%	543	15.8%	56.8%	11097	99.7%	100.0%
Kidney	C64		6911	80.1%	100.0%	438	14.1%	50.4%	7349	86.9%	100.0%
Bladder	C67		5018	66.7%	100.0%	569	17.1%	61.2%	5587	76.6%	100.0%
NHL	C82-C85		12222	96.2%	100.0%	589	17.5%	62.7%	12811	98.3%	100.0%
Leukaemia	C91-C95		6690	78.8%	100.0%	656	19.0%	67.4%	7346	86.9%	100.0%

Statistical power was calculated with type I error rate of 0.05, for Cox regression analysis between diabetes and incident cancer cases with adjustment for age, sex and region.

Table S2. Assessments for residual confounding of fully adjusted associations in meta-analysis of UKB, MWS and CKB

Cancer site	No. of cases (with diabetes)	No. of cases (without diabetes)	HR (95% CI)	P	% Attenuation*	E-value for HR	E-value for CI limit†
Oesophageal AC	161	2034	1.31 (1.11-1.56)	1.87×10^{-3}	51%	1.95	1.45
Oesophageal SCC	184	4477	0.96 (0.83-1.12)	0.61	76%	1.25	1
Stomach	457	6963	1.14 (1.03-1.26)	0.01	19%	1.53	1.21
Colorectum	1658	36321	1.16 (1.10-1.22)	1.68×10^{-8}	22%	1.59	1.43
Liver	640	5343	2.04 (1.87-2.23)	5.89×10^{-57}	19%	3.5	3.15
Pancreas	574	9039	1.62 (1.48-1.77)	3.88×10^{-26}	12%	2.62	2.32
Lung	1700	38250	1.13 (1.07-1.19)	3.49×10^{-6}	-182%	1.51	1.35
Postmenopausal breast	2612	88215	1.05 (1.01-1.10)	0.01	46%	1.29	1.12
Endometrium	745	15615	1.10 (1.02-1.19)	0.01	84%	1.43	1.16
Ovary	345	12151	1.01 (0.91-1.13)	0.86	82%	1.11	1
Prostate	778	10319	0.78 (0.73-0.85)	4.47×10^{-10}	13%	1.87	1.64
Kidney	433	6916	1.27 (1.15-1.41)	3.05×10^{-6}	54%	1.86	1.57
Bladder	352	5235	1.44 (1.29-1.62)	3.00×10^{-10}	12%	2.24	1.9
NHL	442	12369	0.97 (0.88-1.06)	0.48	206%	1.23	1
Leukaemia	338	7008	1.17 (1.05-1.31)	6.18×10^{-3}	34%	1.62	1.27

* From basic model (socioeconomic factors only) to fully adjusted model

† Lower limit of CI for positive associations, and upper limit of CI for inverse associations

Figure S1. Associations of diabetes with 15 cancers in UKB and MWS

The associations were stratified by year of birth, region and sex, and adjusted for socioeconomic factors, smoking, alcohol, physical activity, BMI, waist circumference, early-life age adiposity, hypertension, blood pressure, family history of diabetes and cancer, and female reproductive factors (use of oral contraceptives, HRT, age of menarche, menopausal status and age, parity and age at first birth). Each solid square represents the estimated HR. The horizontal lines indicate 95% CIs and diamonds indicate the combined results of the cohort-specific HRs from UKB and MWS. Associations with FDR-adjusted significant p-values(<0.05) in the meta-analysis of two studies are marked with asterisk on the HR (95% CI).

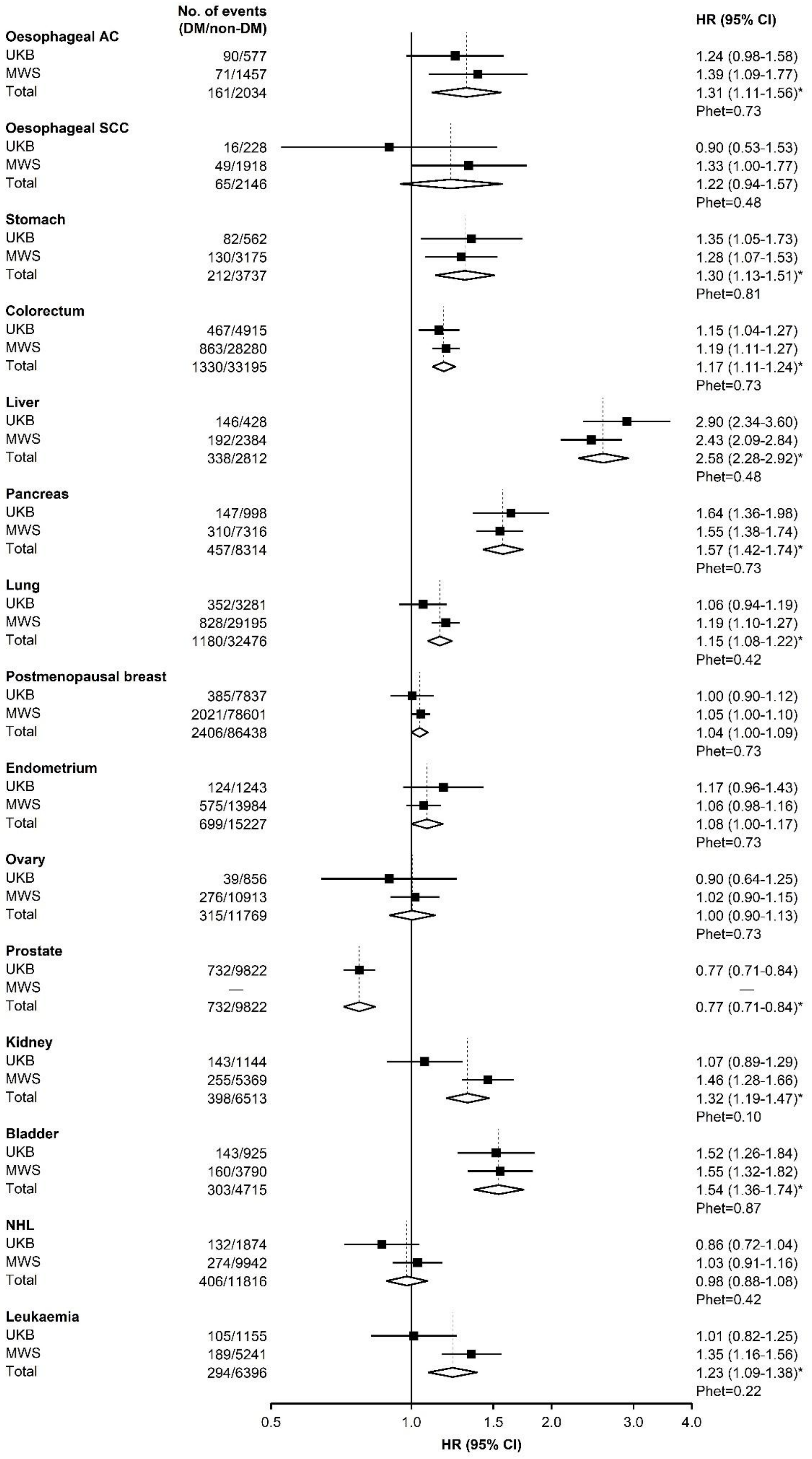

Figure S2. Associations of self-reported and screen-detected diabetes with 15 cancers in UKB and CKB

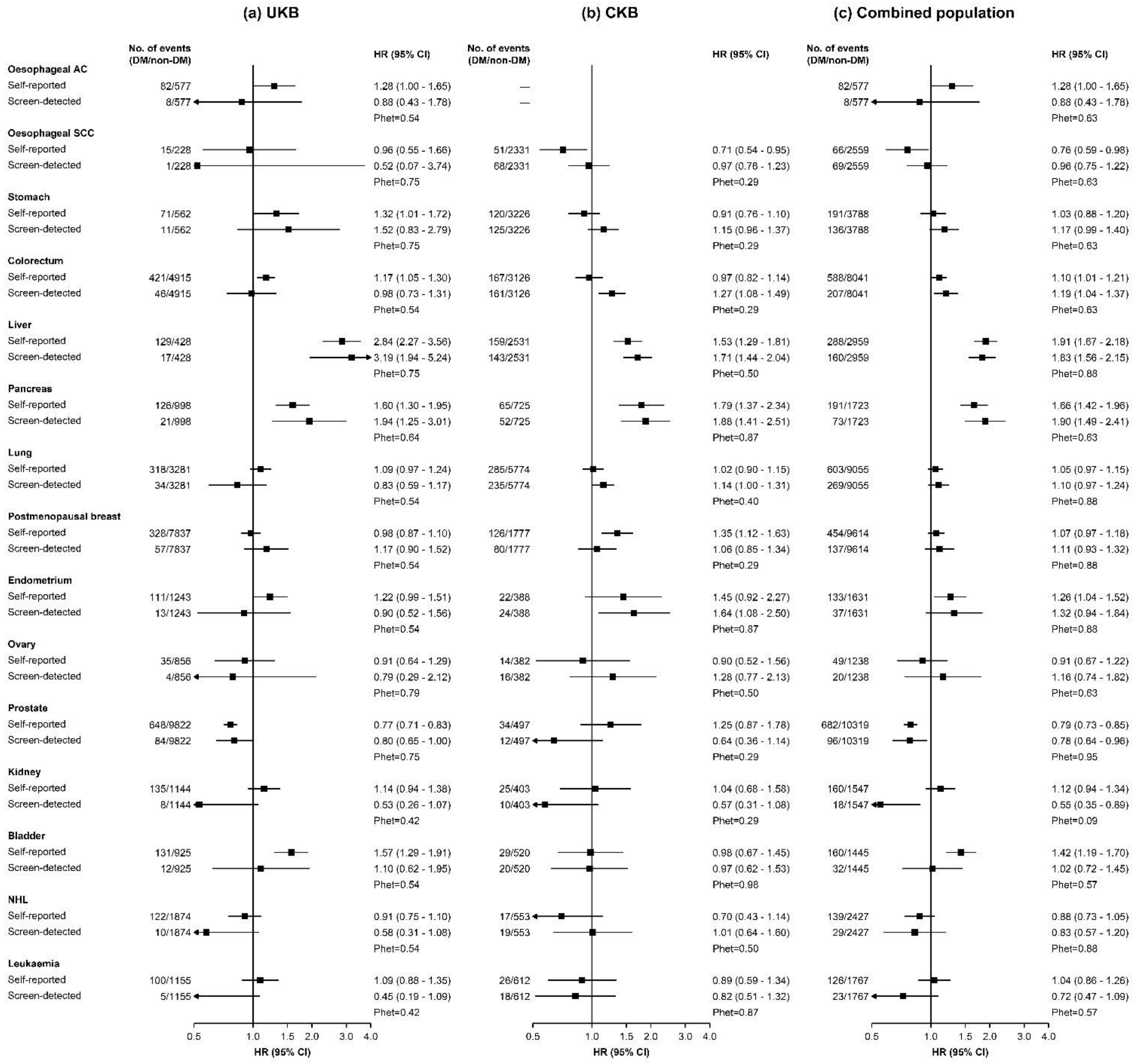

Figure S3. Associations of diabetes with 15 cancers by sex in UKB, MWS and CKB

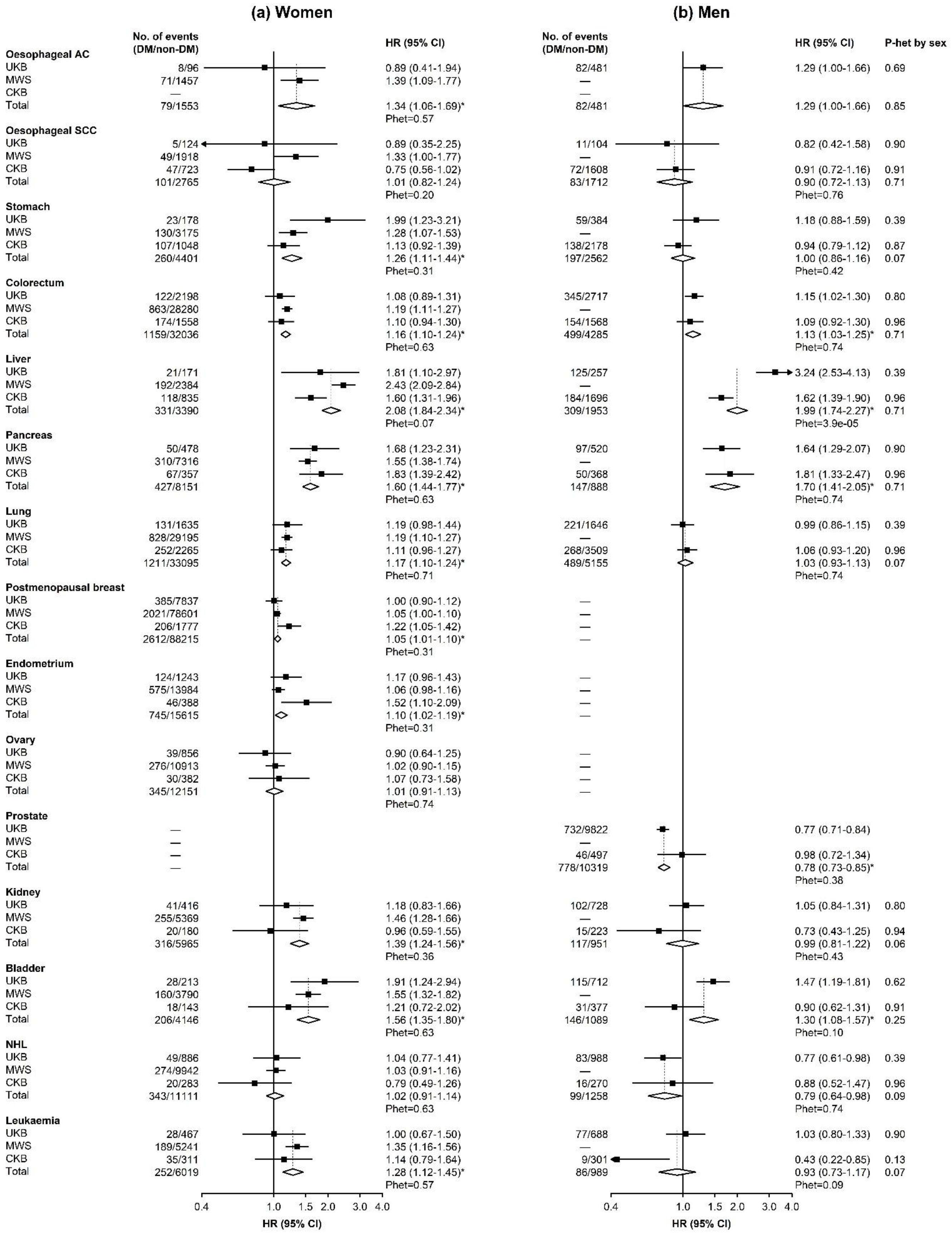

Figure S4. Associations of diabetes with 15 cancer mortality in UK and Chinese studies

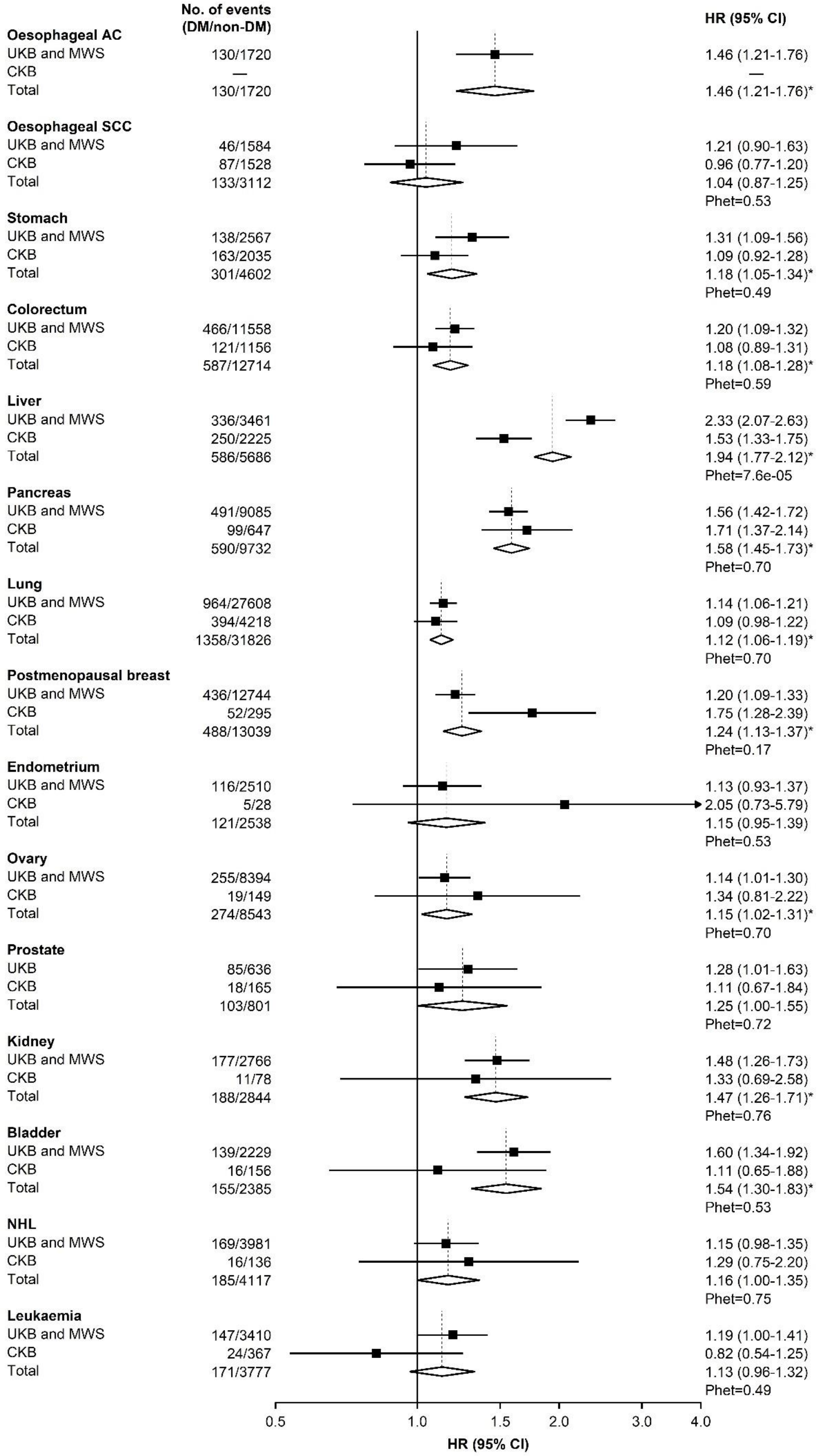

Figure S5. Impact of adjustment for potential confounders on associations of diabetes with 15 cancers in UKB, MWS and CKB

Each group of confounders were adjusted independently from others. The basic model represents the Cox regression model stratified by birth cohort, sex and region, and adjusted for education, Townsend Deprivation index and ethnicity. Adiposity traits include body mass index, waist circumference and early age adiposity in CKB and UKB, and body mass index only in MWS.

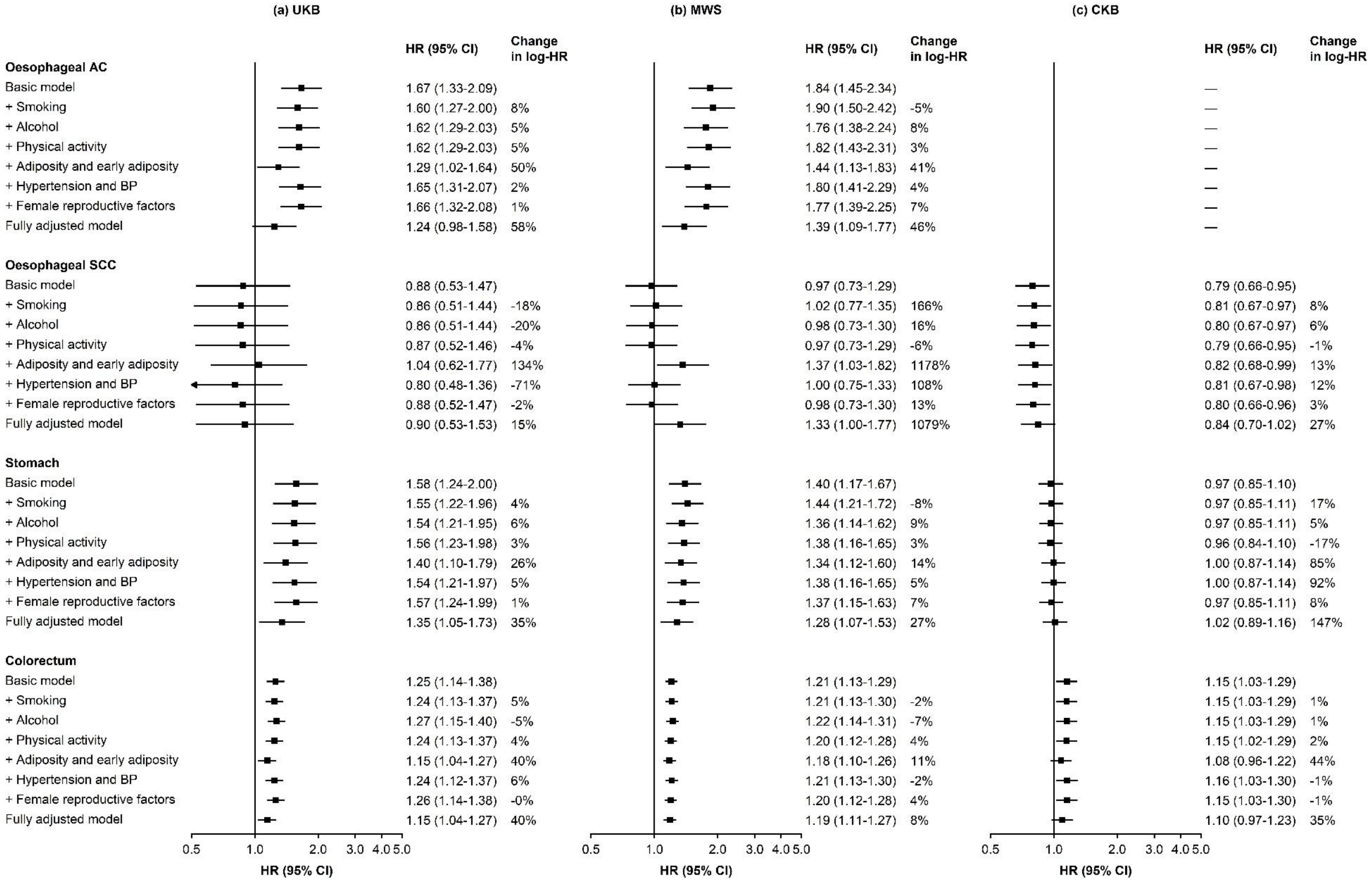

Figure S5 (Cont'd). Impact of adjustment for potential confounders on associations of diabetes with 15 cancers in UKB, MWS and CKB

Each group of confounders were adjusted independently from others. The basic model represents the Cox regression model stratified by birth cohort, sex and region, and adjusted for education, Townsend Deprivation index and ethnicity. Adiposity traits include body mass index, waist circumference and early age adiposity in CKB and UKB, and body mass index only in MWS.

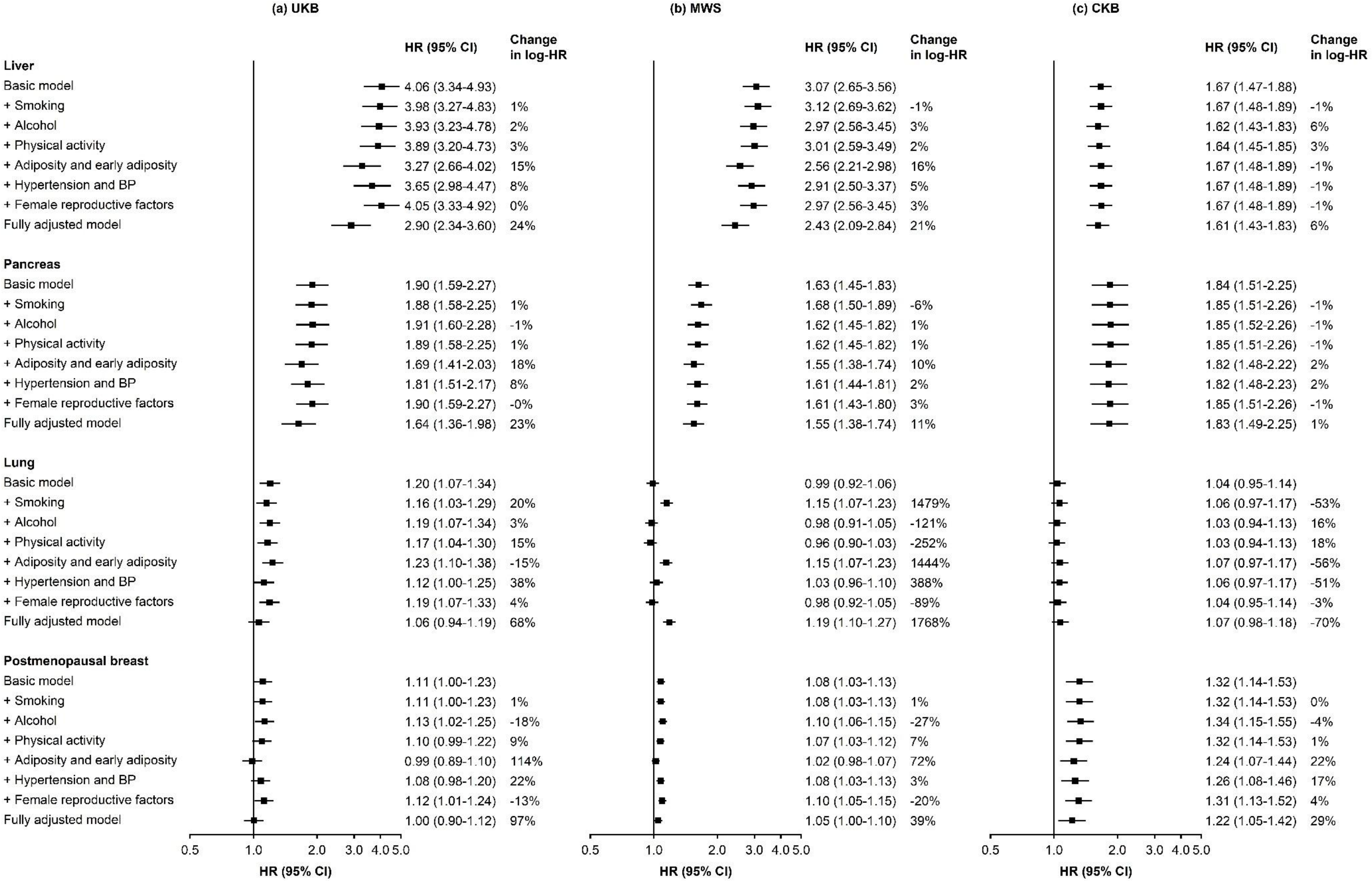

Figure S5 (Cont'd). Impact of adjustment for potential confounders on associations of diabetes with 15 cancers in UKB, MWS and CKB

Each group of confounders were adjusted independently from others. The basic model represents the Cox regression model stratified by birth cohort, sex and region, and adjusted for education, Townsend Deprivation index and ethnicity. Adiposity traits include body mass index, waist circumference and early age adiposity in CKB and UKB, and body mass index only in MWS.

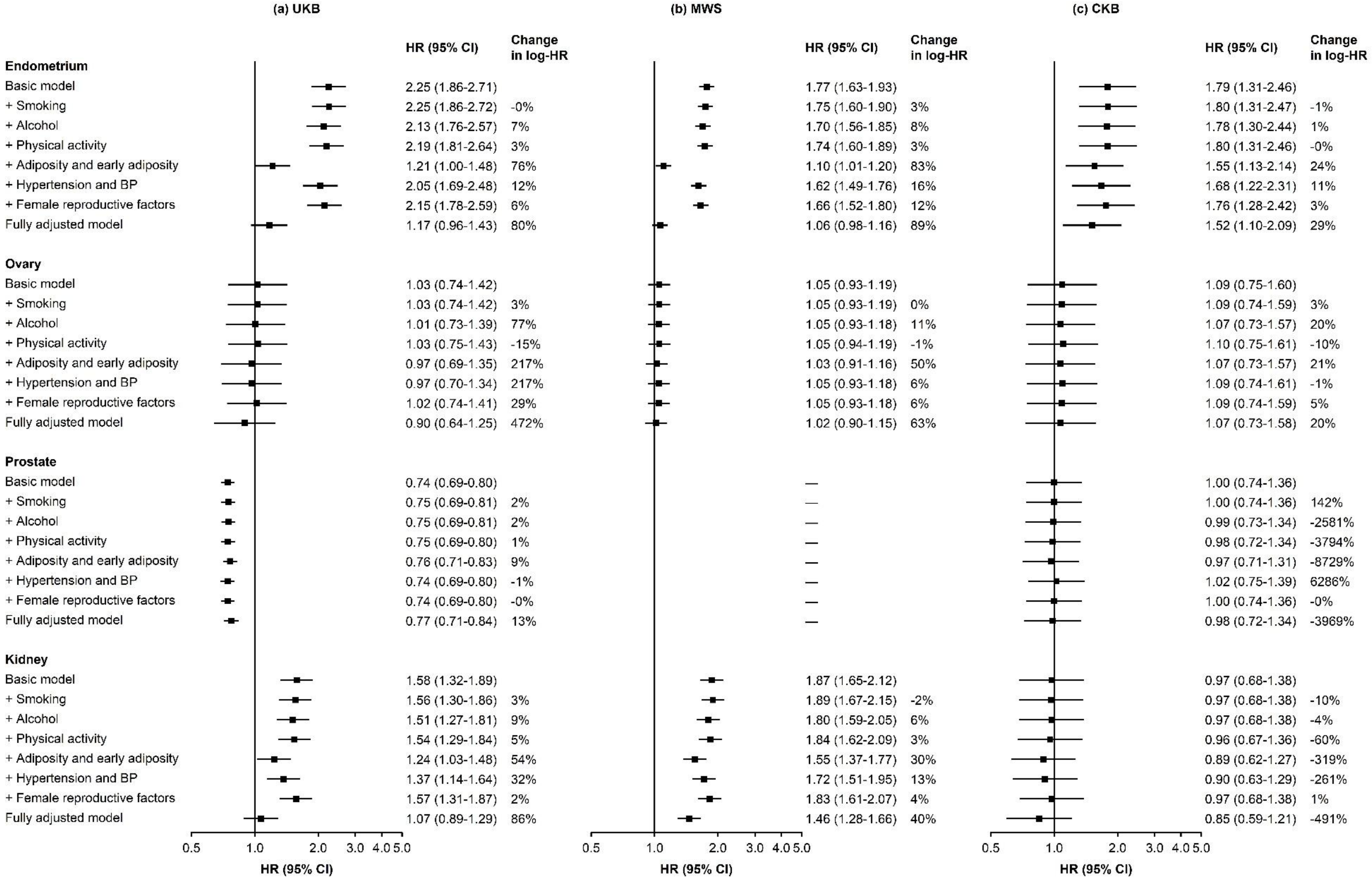

Figure S5 (Cont'd). Impact of adjustment for potential confounders on associations of diabetes with 15 cancers in UKB, MWS and CKB

Each group of confounders were adjusted independently from others. The basic model represents the Cox regression model stratified by birth cohort, sex and region, and adjusted for education, Townsend Deprivation index and ethnicity. Adiposity traits include body mass index, waist circumference and early age adiposity in CKB and UKB, and body mass index only in MWS.

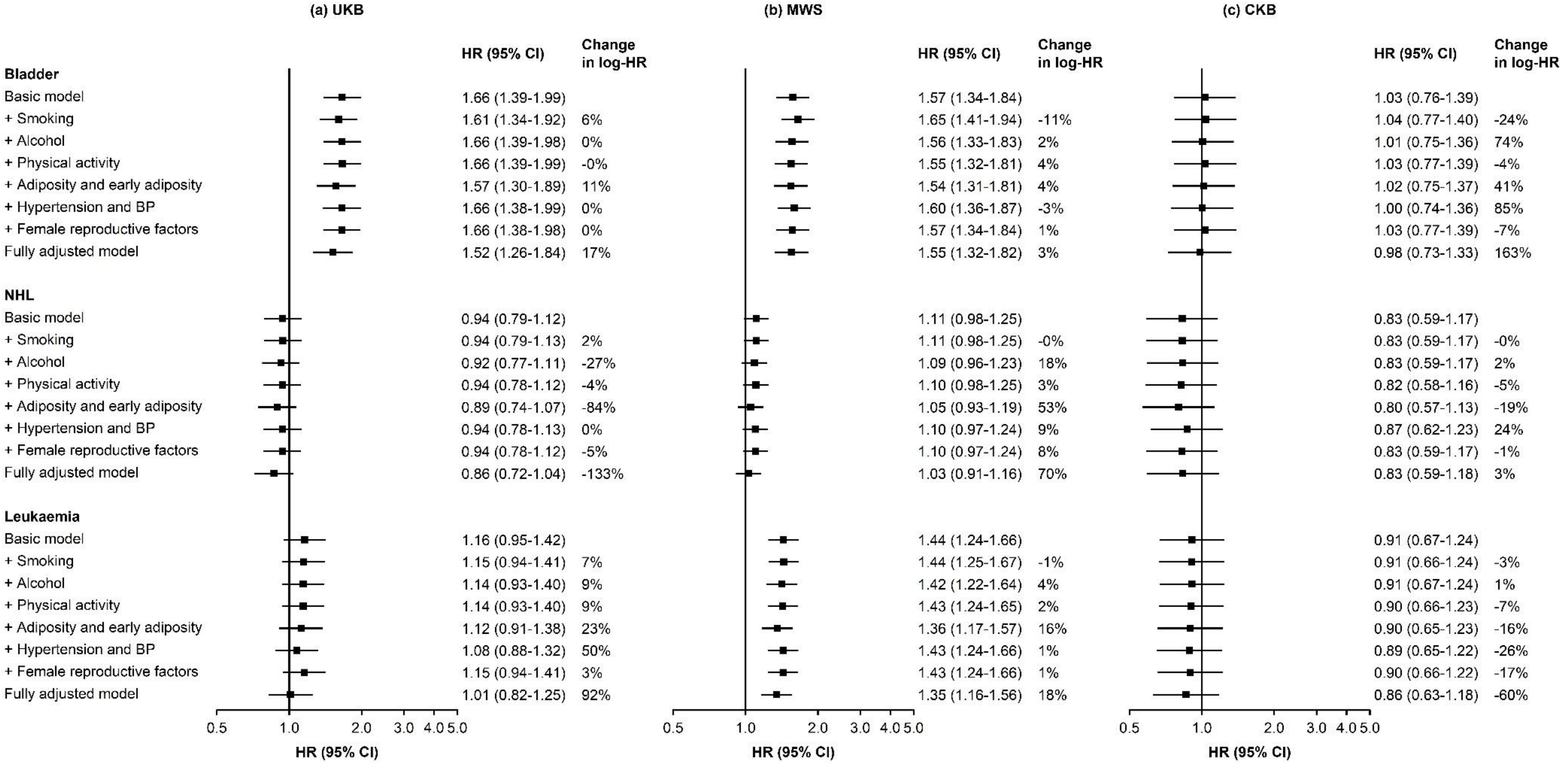

	(a) UKB HR (95% CI)	(a) UKB Change in log-HR	(b) MWS HR (95% CI)	(b) MWS Change in log-HR	(c) CKB HR (95% CI)	(c) CKB Change in log-HR
**Bladder**						
Basic model	1.66 (1.39-1.99)		1.57 (1.34-1.84)		1.03 (0.76-1.39)	
+ Smoking	1.61 (1.34-1.92)	6%	1.65 (1.41-1.94)	-11%	1.04 (0.77-1.40)	-24%
+ Alcohol	1.66 (1.39-1.98)	0%	1.56 (1.33-1.83)	2%	1.01 (0.75-1.36)	74%
+ Physical activity	1.66 (1.39-1.99)	-0%	1.55 (1.32-1.81)	4%	1.03 (0.77-1.39)	-4%
+ Adiposity and early adiposity	1.57 (1.30-1.89)	11%	1.54 (1.31-1.81)	4%	1.02 (0.75-1.37)	41%
+ Hypertension and BP	1.66 (1.38-1.99)	0%	1.60 (1.36-1.87)	-3%	1.00 (0.74-1.36)	85%
+ Female reproductive factors	1.66 (1.38-1.98)	0%	1.57 (1.34-1.84)	1%	1.03 (0.77-1.39)	-7%
Fully adjusted model	1.52 (1.26-1.84)	17%	1.55 (1.32-1.82)	3%	0.98 (0.73-1.33)	163%
**NHL**						
Basic model	0.94 (0.79-1.12)		1.11 (0.98-1.25)		0.83 (0.59-1.17)	
+ Smoking	0.94 (0.79-1.13)	2%	1.11 (0.98-1.25)	-0%	0.83 (0.59-1.17)	-0%
+ Alcohol	0.92 (0.77-1.11)	-27%	1.09 (0.96-1.23)	18%	0.83 (0.59-1.17)	2%
+ Physical activity	0.94 (0.78-1.12)	-4%	1.10 (0.98-1.25)	3%	0.82 (0.58-1.16)	-5%
+ Adiposity and early adiposity	0.89 (0.74-1.07)	-84%	1.05 (0.93-1.19)	53%	0.80 (0.57-1.13)	-19%
+ Hypertension and BP	0.94 (0.78-1.13)	0%	1.10 (0.97-1.24)	9%	0.87 (0.62-1.23)	24%
+ Female reproductive factors	0.94 (0.78-1.12)	-5%	1.10 (0.97-1.24)	8%	0.83 (0.59-1.17)	-1%
Fully adjusted model	0.86 (0.72-1.04)	-133%	1.03 (0.91-1.16)	70%	0.83 (0.59-1.18)	3%
**Leukaemia**						
Basic model	1.16 (0.95-1.42)		1.44 (1.24-1.66)		0.91 (0.67-1.24)	
+ Smoking	1.15 (0.94-1.41)	7%	1.44 (1.25-1.67)	-1%	0.91 (0.66-1.24)	-3%
+ Alcohol	1.14 (0.93-1.40)	9%	1.42 (1.22-1.64)	4%	0.91 (0.67-1.24)	1%
+ Physical activity	1.14 (0.93-1.40)	9%	1.43 (1.24-1.65)	2%	0.90 (0.66-1.23)	-7%
+ Adiposity and early adiposity	1.12 (0.91-1.38)	23%	1.36 (1.17-1.57)	16%	0.90 (0.65-1.23)	-16%
+ Hypertension and BP	1.08 (0.88-1.32)	50%	1.43 (1.24-1.66)	1%	0.89 (0.65-1.22)	-26%
+ Female reproductive factors	1.15 (0.94-1.41)	3%	1.43 (1.24-1.66)	1%	0.90 (0.66-1.22)	-17%
Fully adjusted model	1.01 (0.82-1.25)	92%	1.35 (1.16-1.56)	18%	0.86 (0.63-1.18)	-60%

Figure S6. Associations of diabetes with 15 cancers in UK and Chinese populations, by age at risk

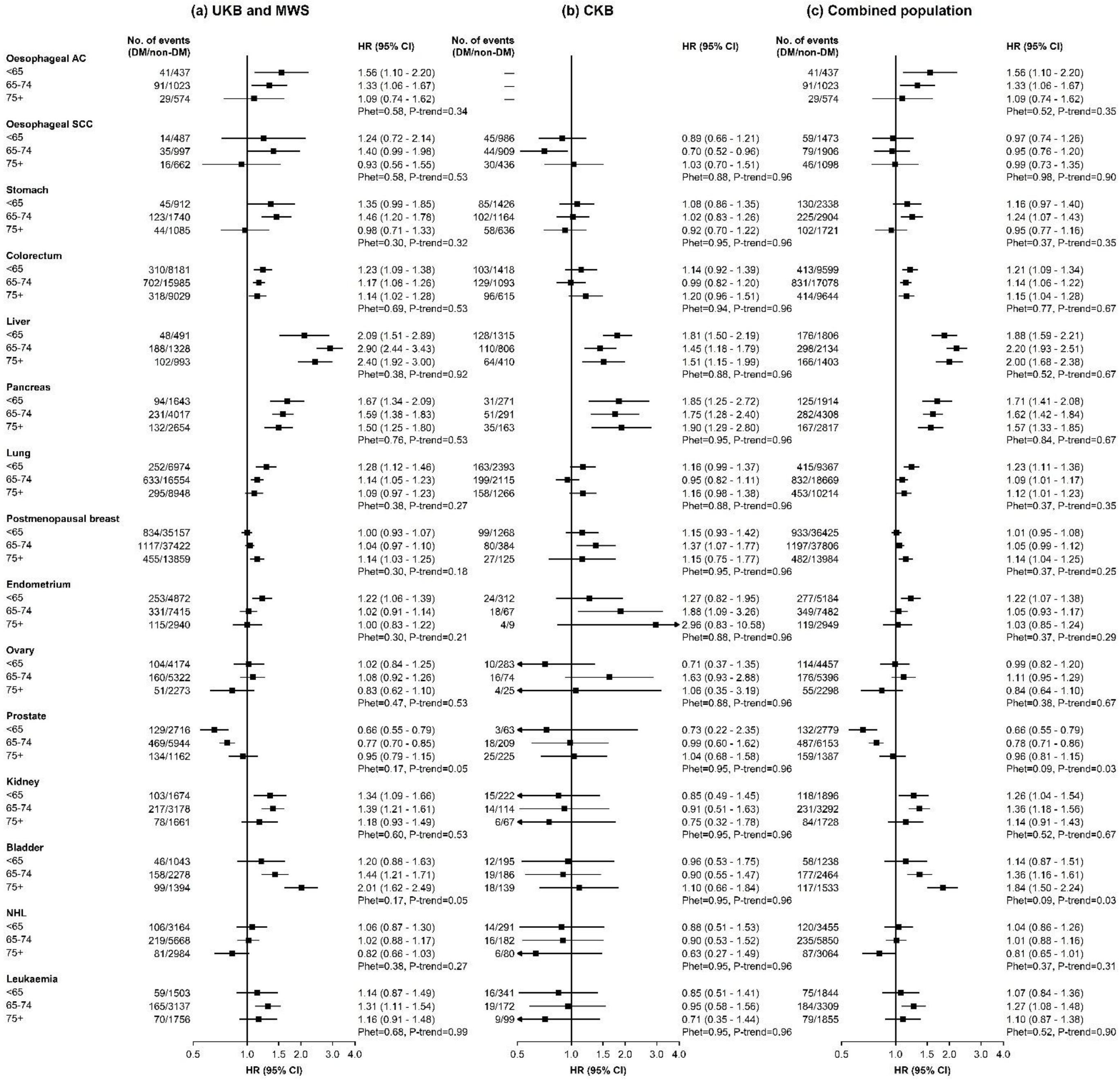

Figure S7. Associations of diabetes with 15 cancers in CKB, by region

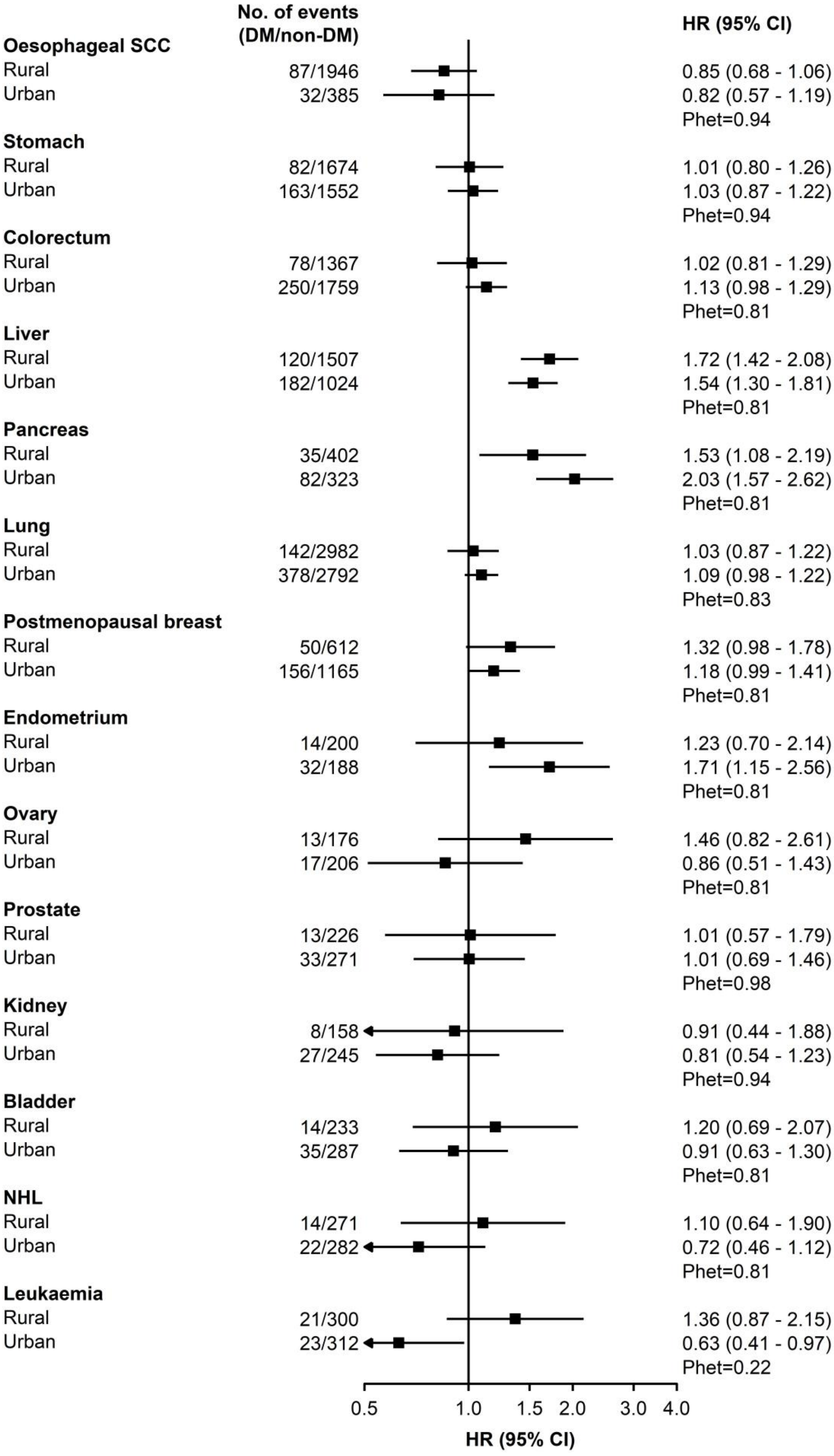

Figure S8. Associations of diabetes with 15 cancers in UK and Chinese populations, by smoking status

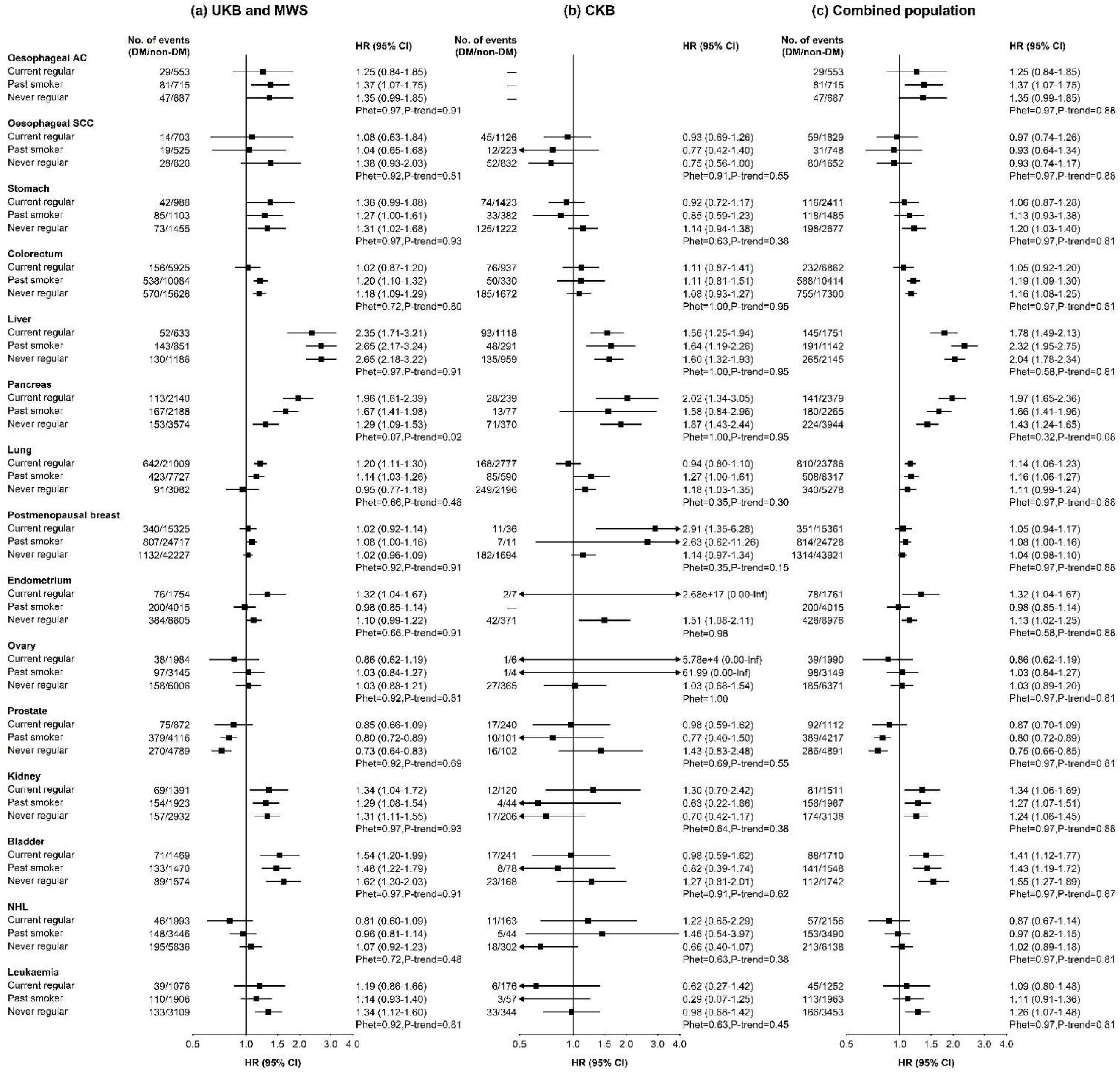

Figure S9. Associations of diabetes with 15 cancers in UK and Chinese populations, by BMI

BMI, kg/m^2

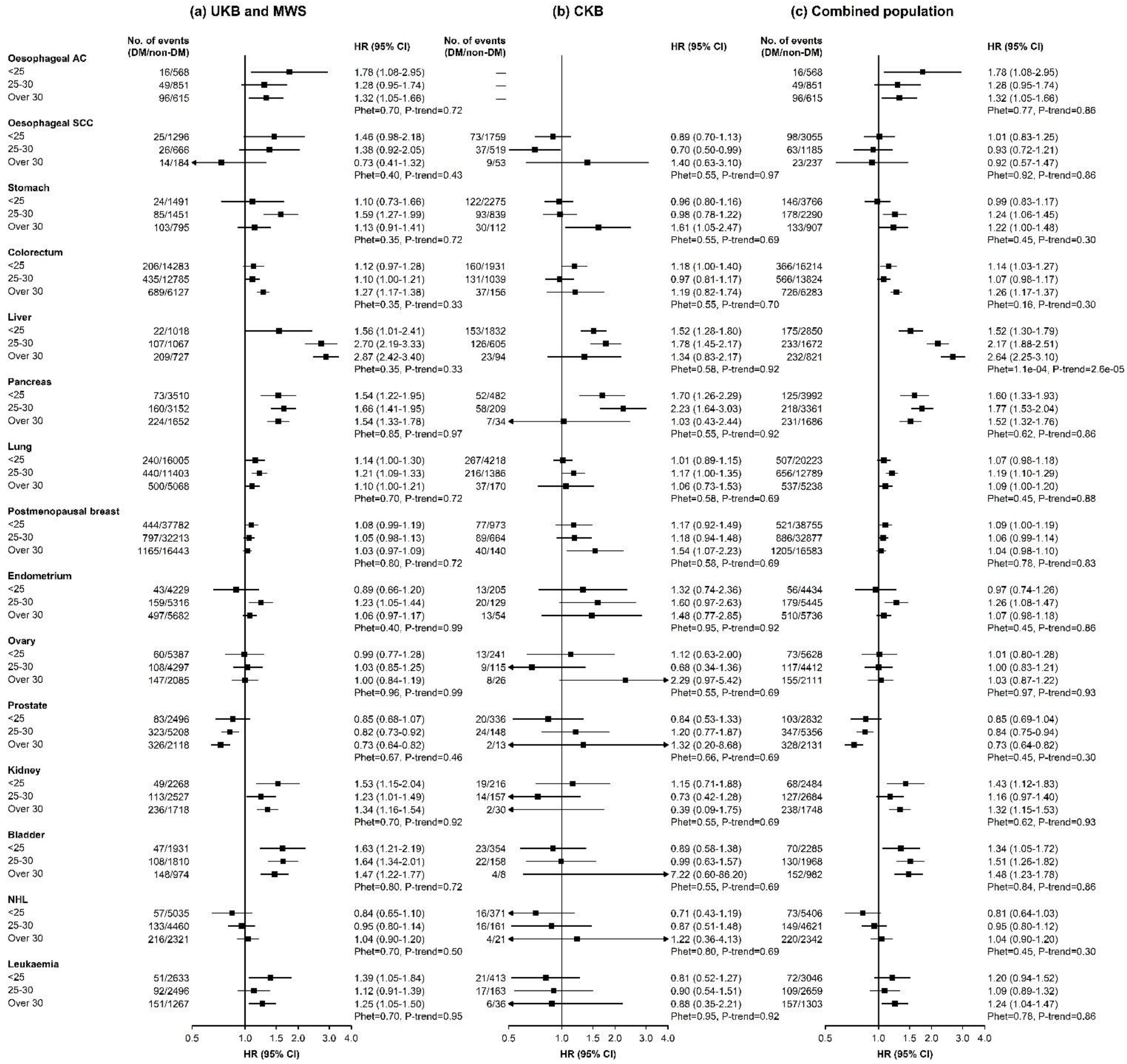

Figure S10. Associations of diabetes with 15 cancers in UK and Chinese populations, by alcohol consumption

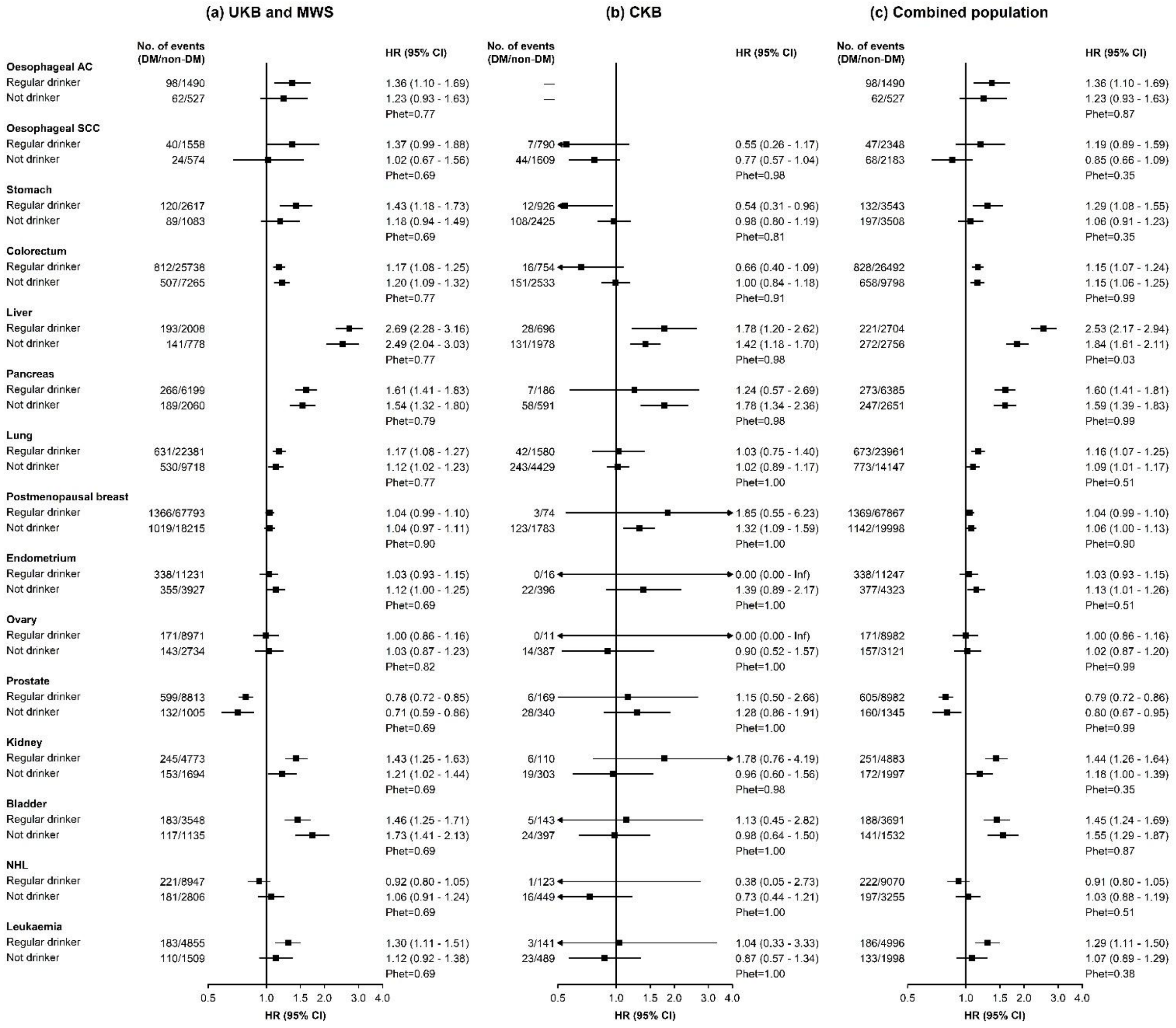

Figure S11. Associations of diabetes with 15 cancers in UK and Chinese populations, by follow-up period

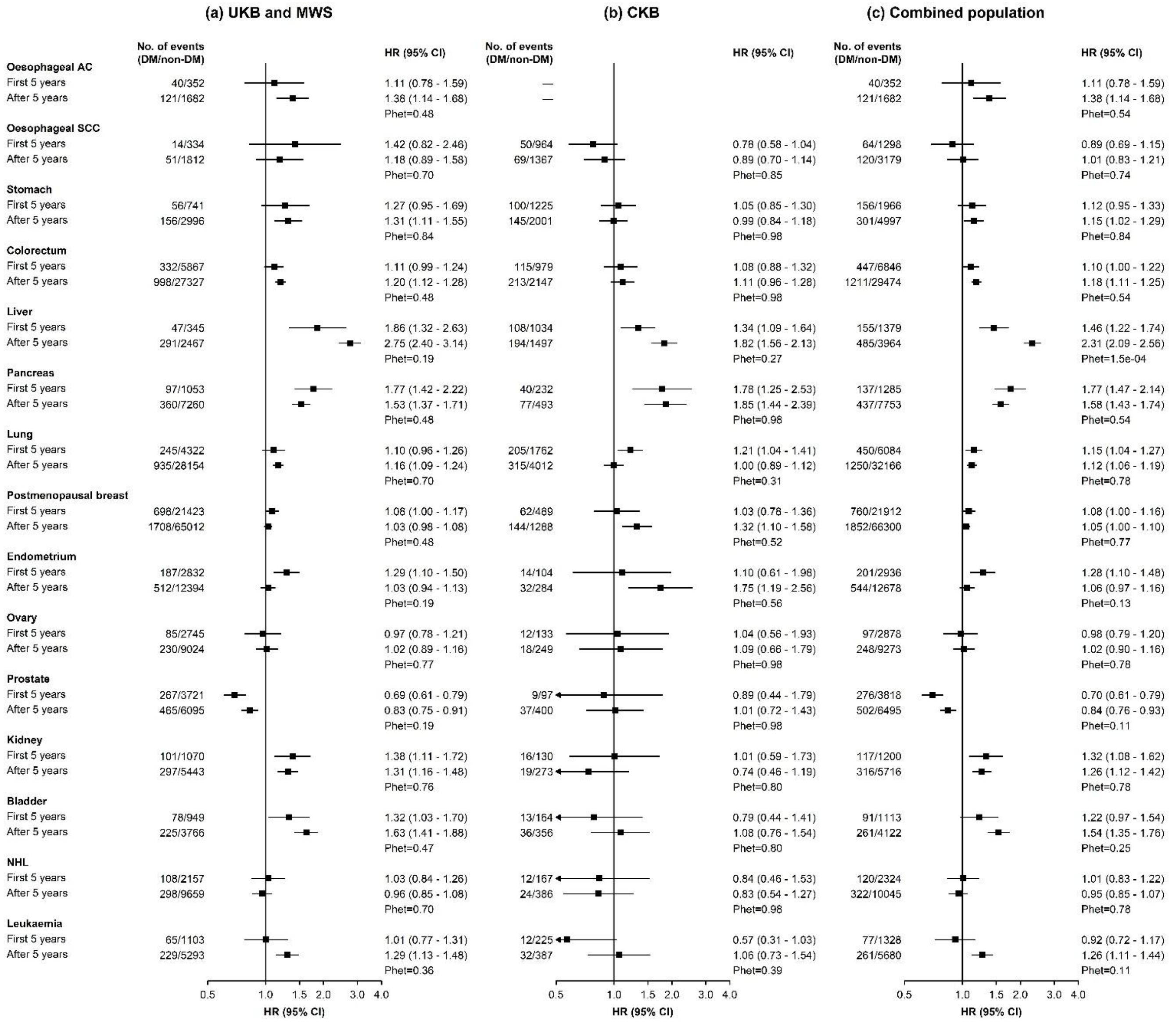

Figure S12. Associations of diabetes with 15 cancers in UK and Chinese populations, by self-reported duration of diabetes

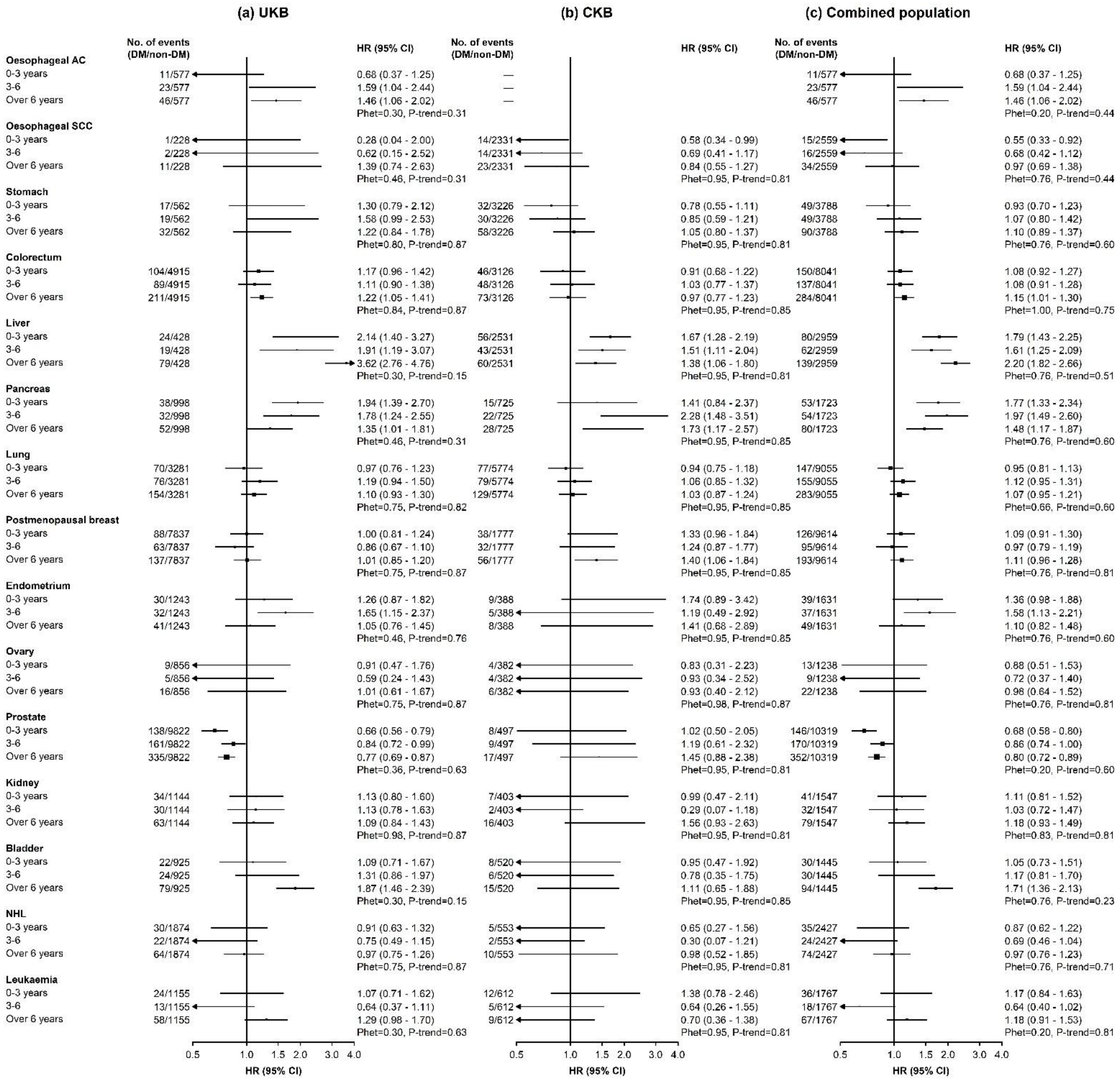

Figure S13. Associations of diabetes with 15 cancers in CKB, with and without exclusion of death certificate only cancer registrations

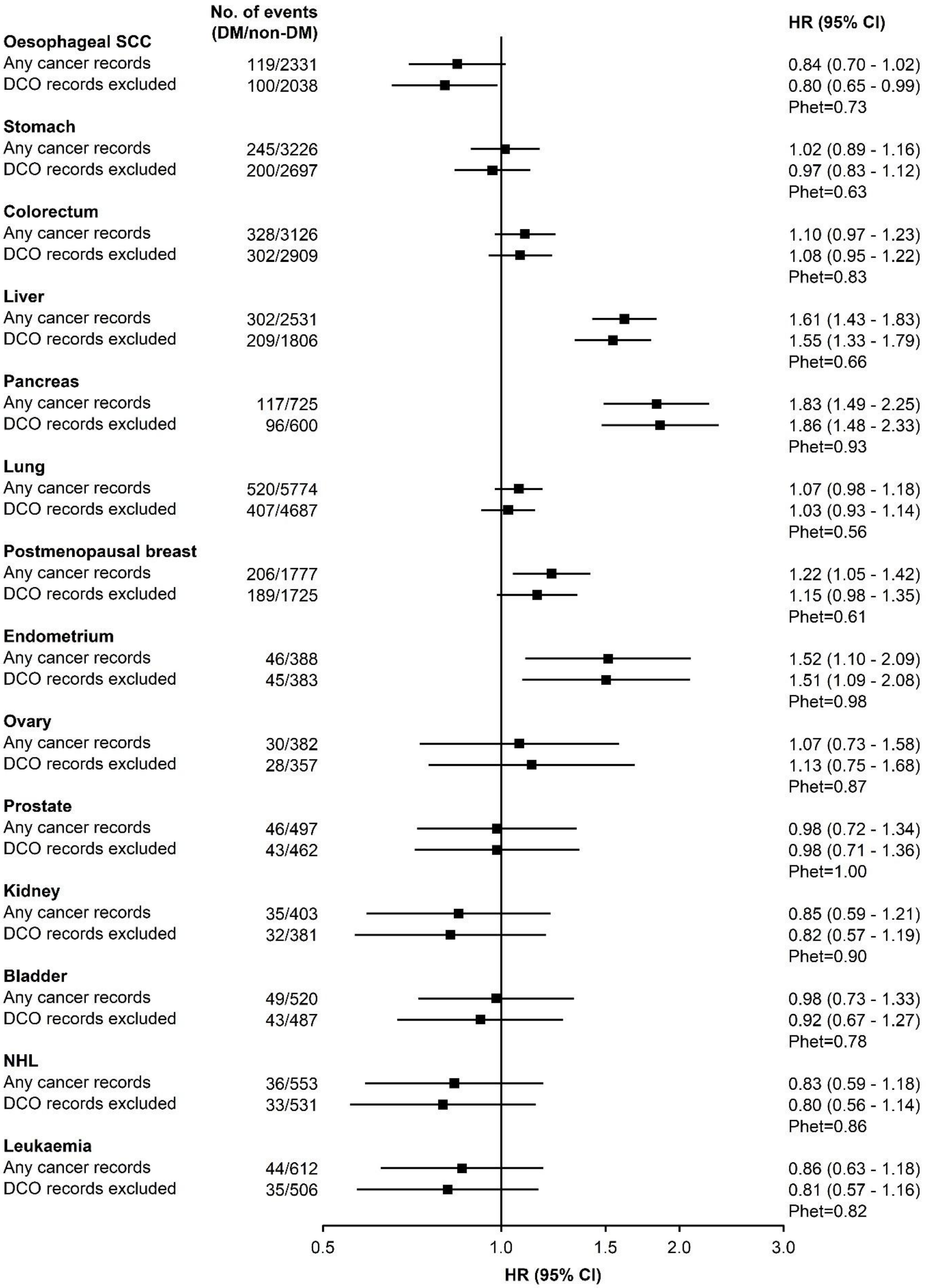

Figure S14. Associations of diabetes with 15 cancers in UKB and CKB, before and after excluding prevalent diabetes diagnosed before age 30

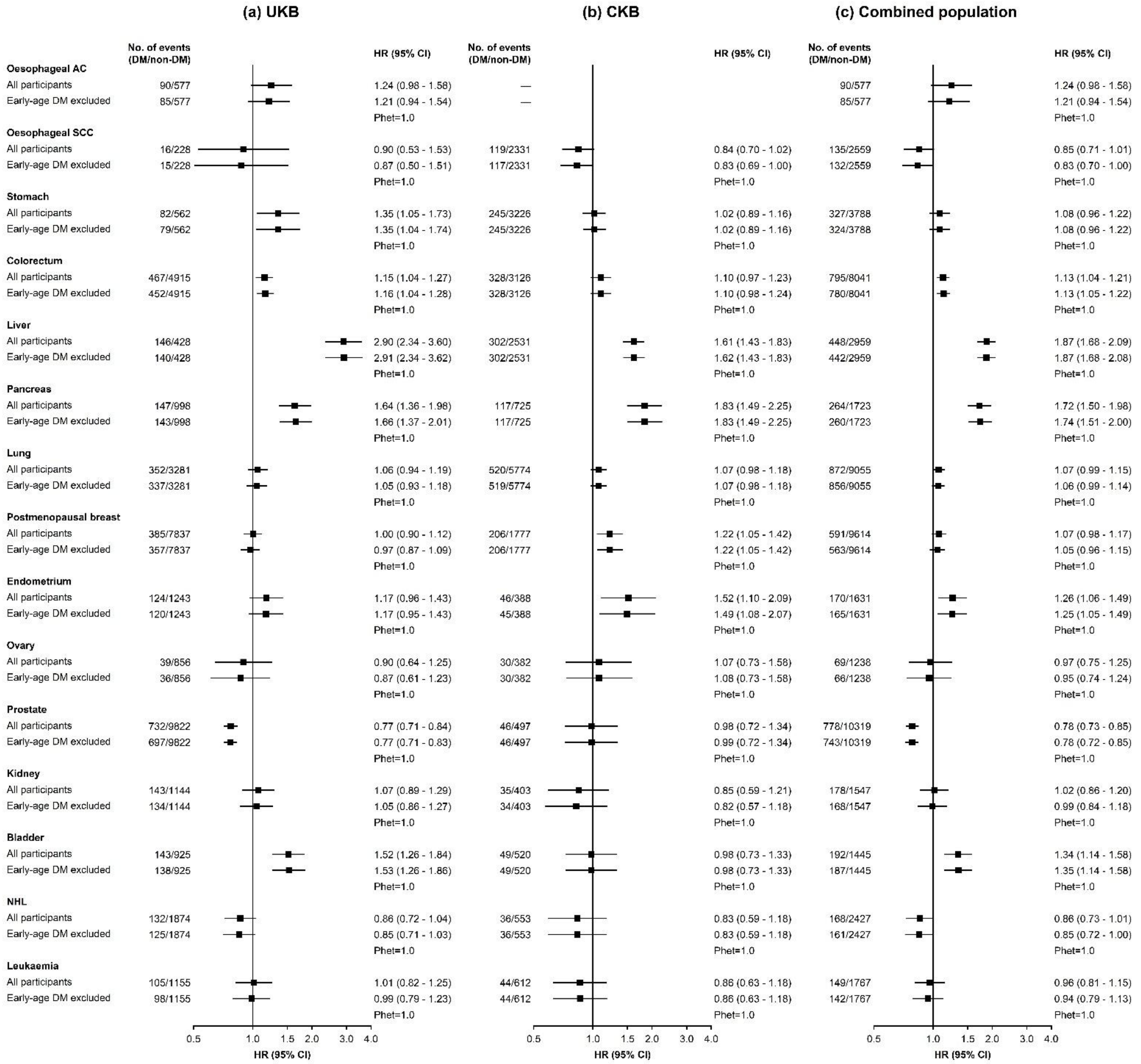

Supplement: djaf154_Supplementary_Data [file djaf154_supplementary_data.pdf]
